# Molecular mechanisms of assembly and TRIP13-mediated remodeling of the human Shieldin complex

**DOI:** 10.1073/pnas.2024512118

**Published:** 2021-02-17

**Authors:** Wei Xie, Shengliu Wang, Juncheng Wang, M. Jason de la Cruz, Guotai Xu, Maurizio Scaltriti, Dinshaw J. Patel

**Affiliations:** ^a^Structural Biology Program, Memorial Sloan Kettering Cancer Center, New York, NY 10065;; ^b^Human Oncology and Pathogenesis Program, Memorial Sloan Kettering Cancer Center, New York, NY 10065

**Keywords:** Shieldin assembly, TRIP13-mediated disassembly of Shieldin, SHLD3–REV7 complex, SHLD2–SHLD3–REV7 complex, SHLD2–SHLD3–REV7–TRIP13 complex

## Abstract

The Shieldin complex, composed of REV7, SHLD1, SHLD2, and SHLD3, protects DNA double-strand breaks (DSBs) to promote nonhomologous end joining. The AAA^+^ ATPase TRIP13 remodels Shieldin to regulate DNA repair pathway choice. Here we report crystal structures of human SHLD3–REV7 binary and fused SHLD2–SHLD3–REV7 ternary complexes, revealing that assembly of Shieldin requires fused SHLD2–SHLD3 induced conformational heterodimerization of open (O-REV7) and closed (C-REV7) forms of REV7. We also report the cryogenic electron microscopy (cryo-EM) structures of the ATPγS-bound fused SHLD2–SHLD3–REV7–TRIP13 complexes, uncovering the principles underlying the TRIP13-mediated disassembly mechanism of the Shieldin complex. We demonstrate that the N terminus of REV7 inserts into the central channel of TRIP13, setting the stage for pulling the unfolded N-terminal peptide of C-REV7 through the central TRIP13 hexameric channel. The primary interface involves contacts between the safety-belt segment of C-REV7 and a conserved and negatively charged loop of TRIP13. This process is mediated by ATP hydrolysis-triggered rotatory motions of the TRIP13 ATPase, thereby resulting in the disassembly of the Shieldin complex.

## Significance

We report on X-ray and cryo-EM structural studies on the assembly of the human Shieldin complex composed of SHLD2, SHLD3, and REV7, as well as its complex with bound TRIP13 toward understanding the principles underlying TRIP13-mediated disassembly of Shieldin. Our studies identify a conformational heterodimeric alignment of open (O) and closed (C) conformers of REV7 when bound to a fused SHLD2–SHLD3 construct. The AAA^+^ ATPase TRIP13 captures the N terminus of C-REV7 (REV^NT^) within its central hexameric channel, with the rotatory motion associated with sequential ATP hydrolysis within individual TRIP13 subunits. This facilitates the stepwise pulling of the REV7^NT^ through the central channel, resulting in initial disassembly of C-REV7 followed by dissociation of the Shieldin complex.

DNA double-strand breaks (DSBs) represent one of the most damaging lesions to the integrity of double helical DNA (1). DSBs are repaired either by error-prone nonhomologous end joining (NHEJ) or by error-free homology-directed repair (HDR) (2). The decision-making point controlling these two DSB repair pathways involves the initiation of DNA termini resection (3, 4). Briefly, the tumor suppressor BRCA1 promotes HDR by enhancing DNA end resection, since HDR requires 3′ DNA overhangs (5). By contrast, a chromatin-binding protein 53BP1 counteracts DSB resection and facilitates NHEJ that requires unresected DNA ends (6–8). How 53BP1 suppresses DSB resection has long been enigmatic, but recent studies have highlighted the contribution of the 53BP1–RIF1–Shieldin pathway to this process (9, 10). The Shieldin complex acts as the key downstream effector of 53BP1; it not only binds and shields single-stranded DNA ends but also mediates CST- and Polα-dependent fill-ins of DNA breaks (10–14). Notably, loss-of-function mutations of Shieldin alleviate the HDR defect of BRCA1-mutated cells, thereby restoring resistance to poly(ADP ribose) polymerase inhibition (PARPi) (13–16). Thus, elucidation of mechanistic insights into the role of the Shieldin complex is essential to combat chemotherapeutic resistance and to uncover anticancer drug targets.

Human Shieldin consists of four subunits, REV7 (also known as MAD2L2 or MAD2B, 211 residues), SHLD1 (205 residues), SHLD2 (835 residues), and SHLD3 (250 residues) (14). As the first identified member of the Shieldin complex (17, 18), REV7 is composed entirely of a HORMA domain, acting as an interaction module in a broad array of cellular pathways (19–22). SHLD3 and REV7 form a proximal subcomplex working as a localization module in the 53BP1–RIF1–REV7 axis (13). SHLD2 is the scaffold that bridges SHLD3–REV7 and SHLD1, as well as binds single-stranded DNA ends via its C-terminal oligonucleotide/oligosaccharide-binding fold (OB-fold) domain (23–25). Recently, a crystal structure of the SHLD3 fragment–REV7(R124A) binary complex confirms that monomeric REV7 capitalizes on its stereotypical “safety belt,” a common structural feature of HORMA family proteins, to encircle SHLD3 (26). Further, another crystal structure of Rev7 bound to fragments of SHLD2 and SHLD3 has provided insights into the assembly of this Shieldin trimeric subcomplex (27). Of note, a recent report identified that AAA^+^ ATPase TRIP13 is a negative regulator of Shieldin (28). TRIP13 forms a hexamer to drive the REV7-mediated disassembly of Shieldin in an adenosine triphosphate (ATP)-dependent manner, thereby releasing the DSB DNA ends for resection to promote HDR (29–31). The overexpression of TRIP13 causes PARPi resistance and correlates with poor survival of patients (28, 32). Thus, disrupting the Shieldin–TRIP13 interaction represents an ideal strategy to potentiate the clinical effectiveness of PARPi (28, 33).

As part of our effort to decipher the molecular mechanisms of assembly and TRIP13-mediated remodeling of the human Shieldin complex, we have solved X-ray and cryogenic electron microscopy (cryo-EM) structures of a series of Shieldin complexes of increasing size and complexity, culminating in the complex of Rev7, a fused SHLD2–SHLD3 fragment and TRIP13. Our studies highlight the principles underlying how TRIP13 facilitates the disassembly of REV7 in the context of the SHLD2–SHLD3–REV7 complex.

## Results

### Crystal Structure of the SHLD3_35–58_–REV7 Monomer Complex.

**Fig. 1.**
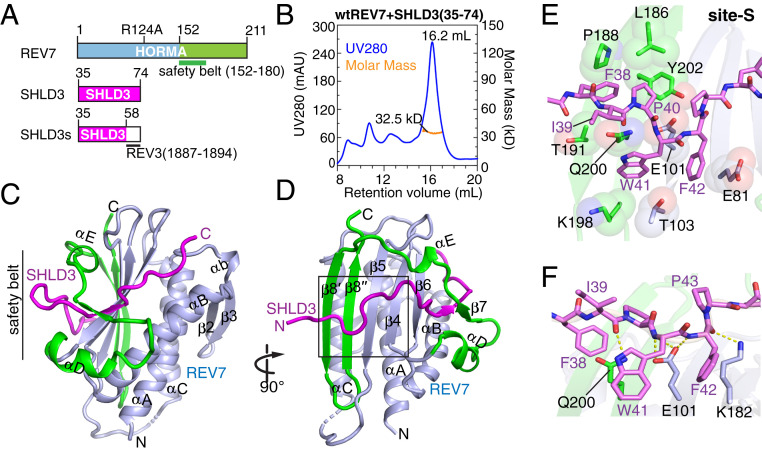
Crystal structure of SHLD3s–REV7 monomer complex reveals safety-belt topology and site-S interface. (*A*) Schematic drawing of human REV7 and SHLD3s fusion protein. The safety-belt segment of REV7 spans residues 152 to 180. (*B*) Purification of the SHLD3 (35 to 74)–REV7 monomer complex on an S200 gel filtration column. The major peak exhibited a mol. wt. = 32.5 kDa by SEC-MALS. (*C* and *D*) Two views of the overall structure of the SHLD3s–REV7 monomer complex. The N- and C-terminal halves of REV7 monomer are colored in light blue and green, respectively, while SHLD3s is colored in magenta. A black box highlights the site-S region in *D*. (*E* and *F*) Hydrophobic (*E*) and hydrogen bonding (*F*) interactions involving site-S. As shown in *E*, the bulky side chain of SHLD3 Phe-38 wedges into the hydrophobic pocket lined by REV7 residues Leu-186, Pro-188, Thr-191, and Tyr-202, while SHLD3 Trp-41 and Phe-42 stack tightly with the side chains of Glu-81, Glu-101, Thr-103, Lys-198, and Gln-200 of REV7. As shown in *F*, the backbones of SHLD3 Ile-39, Trp-41, Phe-42, and Pro-43 further interacts with the side chains of REV7 residues Glu-101, Lys-182, and Leu-173, and Ala-174 by hydrogen bonding.

To investigate the initial step in the assembly of Shieldin, we first generated a complex by coexpressing full-length REV7 (R124A mutant) and the 35- to 74-residue fragment of SHLD3 (Fig. 1*A*). REV7 Arg-124 was mutated to Ala to improve crystallization, as reported previously (34). The major peak on an S200 gel filtration column following formation of the SHLD3_35–74_–REV7 complex exhibited a molar mass of 32.5 kDa measured by size-exclusion chromatography coupled with in-line multiangle light-scattering analysis (SEC-MALS), close to the theoretical molecular mass (28.9 kDa) of the monomer (Fig. 1*B*). Attempts at crystallization of this complex were unsuccessful. Next, we coexpressed full-length REV7 (R124A mutant) and a fusion protein that contains SHLD3 (residues 35 to 58) and a short stabilizing REV3 sequence (residues 1887 to 1894) (labeled SHLD3s, Fig. 1*A*). REV3 is the catalytic subunit of translesion DNA polymerase ζ. REV7 acts as the accessory subunit of DNA polymerase ζ by directly binding REV3. The reported structure of the REV7–REV3 complex implied that the additional short REV3 sequence could potentially enhance complex formation (34, 35). We then determined the crystal structure of this SHLD3s–REV7 monomer complex at 2.7-Å resolution (Fig. 1 *C* and *D*; X-ray statistics in *SI Appendix*, Table S1). There are two molecules in the asymmetric unit (*SI Appendix*, Fig. S1*A*) and the bound SHLD3s can be readily traced into its density in the complex (*SI Appendix*, Fig. S1*B*).

REV7 adopts a closed conformation (designated C-REV7) in this SHLD3s–REV7 monomer complex, such that the REV7-binding motif (RBM) of SHLD3 is threaded through REV7, revealing the typical safety-belt architecture (*SI Appendix*, Fig. S2 *A* and *B*; boxed segment on *SI Appendix*, Fig. S1 *A*, *Lower*). The observed intermolecular hydrophobic and hydrogen bonding contacts within the safety-belt segment of the SHLD3s–REV7 monomer complex are shown in *SI Appendix*, Fig. S2 *C* and *D*, respectively, and details are listed in the figure captions.

Interestingly, the structure of the SHLD3s–REV7 monomer complex revealed an unanticipated additional interface, termed “site-S” (boxed region, Fig. 1*D* and *SI Appendix*, Fig. S3*A*), where the hydrophobic residue cluster (38-FIPWF-42) of SHLD3 wraps over the surface of the REV7 β-sheet region (composed of β4 to β6, β8′, and β8′′), with details of intermolecular contacts listed in the captions to Fig. 1 *E* and *F*.

### Generation of Complexes of REV7 Bound to Fused Fragments of SHLD2 and SHLD3.

**Fig. 2.**
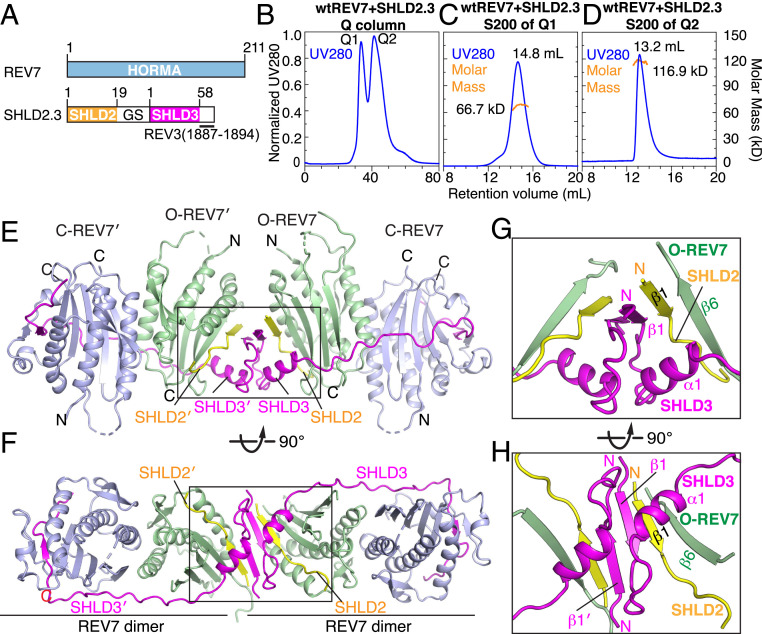
Crystal structure of SHLD2.3–REV7_4_ complex. (*A*) Schematic drawing of human REV7 and SHLD2.3 fusion protein. (*B*) Q column purification of the complex SHLD2.3 bound to REV7 yields two peaks labeled Q1 and Q2. (*C*) Size exclusion S200 purification of peak labeled Q1 and measurement of a 66.7-kDa molar mass for the major peak by SEC-MALS. (*D*) Size exclusion S200 purification of peak labeled Q2 and measurement of a 116.9 kDa molar mass by SEC-MALS. (*E* and *F*) Two views of the overall structure of SHLD2.3–REV7_4_ complex. C-REV7, light blue; O-REV7, green; SHLD3, magenta; SHLD2, yellow. (*G* and *H*) Expanded views of the boxed segment in *E* (see *G*) and *F* (see *H*) of the complex highlighting the dimeric interface whereby SHLD2 β1–SHLD3 β1–REV7 β6 segments form a pair of β-sheets.

Though REV7 was shown above to be a monomer in the SHLD3s–REV7 complex, it also adopts a dimeric alignment in the structure of translesion DNA polymerase ζ (36, 37). To test whether REV7 can adopt a dimeric alignment in its complexes with SHLD peptides, we next designed a fusion protein containing an SHLD2 fragment (residues 1 to 19) fused through a Gly–Ser linker to an SHLD3 fragment (residues 1 to 58) and a C-terminal fragment of REV3 (residues 1887 to 1894) (labeled SHLD2.3, Fig. 2*A*). We observed that the SHLD2.3–REV7 complex yielded two peaks (labeled Q1 and Q2) on a Hitrap Q column (Fig. 2*B*). The fraction from peak Q1 eluted at 14.8 mL on an S200 gel filtration column, exhibiting a molar mass of 66.7 kDa by SEC-MALS, indicative of formation of a SHLD2.3–Rev7 dimer complex (labeled SHLD2.3–Rev7_2_ complex, Fig. 2*C*). The fraction from peak Q2 eluted at 13.2 mL on an S200 gel filtration column, exhibiting a molar mass of 116.9 kDa by SEC-MALS, indicative of formation of a SHLD2.3–REV7 tetramer complex (labeled SHLD2.3–REV7_4_ complex, Fig. 2*D*). These results indicate that the designed SHLD2.3 fusion protein can induce both dimerization and tetramerization of REV7 in solution.

### Crystal Structure of the SHLD2.3–Rev7_4_ Complex.

We attempted to crystallize the SHLD2.3–REV7 complexes corresponding to peaks Q1 (Fig. 2*C*) and Q2 (Fig. 2*D*) and were only successful for the latter tetrameric complex. The structure of this SHLD2.3–Rev7_4_ complex was solved at 3.8-Å resolution (X-ray statistics in *SI Appendix*, Table S1) and is shown in two orientations (Fig. 2 *E* and *F*). The electron density could be traced for all components of this complex (*SI Appendix*, Fig. S4*A*), including SHLD2 (*SI Appendix*, Fig. S4*B*) and SHLD3 (*SI Appendix*, Fig. S4*C*). The crystallographic asymmetric unit contains one SHLD2.3–REV7_4_ complex composed of a pair of REV7 conformational heterodimers connected by a pair of head-to-head aligned SHLD2.3–SHLD2.3 β-sheets (Fig. 2 *G* and *H*).

**Fig. 3.**
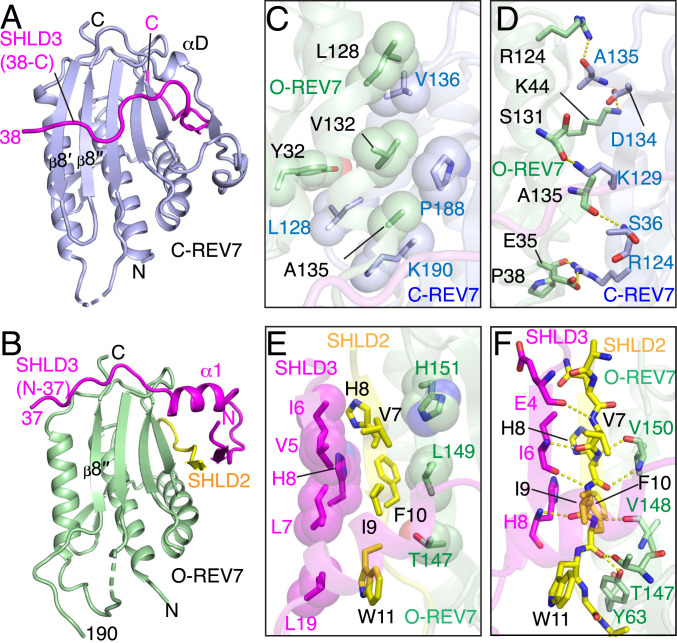
Comparison of closed C-REV7 and open O-REV7 conformations in the SHLD2.3–REV7_4_ complex and intermolecular contacts. (*A* and *B*) Closed C-REV7 (*A*) and open O-REV7 (*B*) conformations in the SHLD2.3–REV7_4_ complex. (*C* and *D*) Dimeric interface between C-REV7 and O-REV7 in the SHLD2.3–REV7_4_ complex is mediated by multiple intermolecular hydrophobic (*C*) and hydrogen bonding (*D*) interactions. Trp-32, Leu-128, Val-132, and Ala-135 of O-REV7 build up a hydrophobic core with Leu-128, Vla-136, Pro-188, and Lys-190 of C-REV7 (*C*). Both Arg-124 of O-REV7 and C-REV7 form hydrogen bonds with Ala-135 of C-REV7 and Glu-35 of O-REV7, respectively, highlighting the importance of Arg-124 that is shown in previous reports. Lys-44, Ser-131, and Ala-135 of O-REV7 form additional hydrogen bonds with Asp-134, Lys-129, and Ser-36, respectively (*D*). (*E* and *F*) SHLD2–SHLD3 alignment in the O-REV7 is mediated by multiple intermolecular hydrophobic (*E*) and hydrogen bonding (*F*) interactions. Thr-147, Leu-149, and His-151 of O-REV7 and Val-5, Ile-6, Leu-7 His-8, and Leu-19 of SHLD3 build a hydrophobic core with Val-7, His-8, Ile-8, Phe-10, and Trp-11 of SHLD2 (*E*). The β-sheet is further stabilized by multiple backbone hydrogen bonds, while the backbone carbonyl oxygen of SHLD2 Phe-10 is recognized by the side chains of REV7 Tyr-63 and Thr-147 by hydrogen bonding (*F*).

In each REV7 conformational heterodimer (Fig. 2 *E* and *F*), one REV7 adopts an open (O-REV7, Fig. 3*A*) while the other exhibits a closed (C-REV7, Fig. 3*B*) conformation. The SHLD2.3 fusion protein serves as a strap with its N and C termini binding O-REV7 and C-REV7, respectively (Fig. 2 *E* and *F*), therby strengthening the conformational heterodimerization of REV7. The 936 Å^2^ dimeric interface between O-REV7 and C-REV7 is further mediated by multiple intermolecular hydrophobic (Fig. 3*C*) and hydrogen bonding (Fig. 3*D*) interactions. Notably, Arg-124 of O-REV7 forms hydrogen bonds with Ala-135 of C-REV7, while Arg-124 of C-REV7 forms hydrogen bonds with Glu-35 of O-REV7, highlighting the importance of Arg-124, consistent with previous reports (27, 34).

The C-REV7 forms the typical safety-belt including an intact β8′/β8″ hairpin (Fig. 3*A*), identical to that observed in the SHLD3s–REV7 complex (Fig. 1*D*), while O-REV7 lacks both the safety belt and the β8′ strand (Fig. 3*B*). The β1 strand of SHLD2 is positioned between the β6 strand of O-REV7 (antiparallel alignment) and the β1 strand of SHLD3 (parallel alignment) as shown in Fig. 2 *G* and *H*, with this sandwiched β-sheet topology stabilized by hydrophobic (Fig. 3*E*) and hydrogen bonding (Fig. 3*F*) interactions. The above results provide structural insights into SHLD2–SHLD3 mediated REV7 conformational heterodimerization involving C-REV7 and O-REV7 subunits, which constitute an essential initial step in the Shieldin assembly.

### Reconstitution of Hexameric TRIP13 Bound to the Shieldin Complex.

**Fig. 4.**
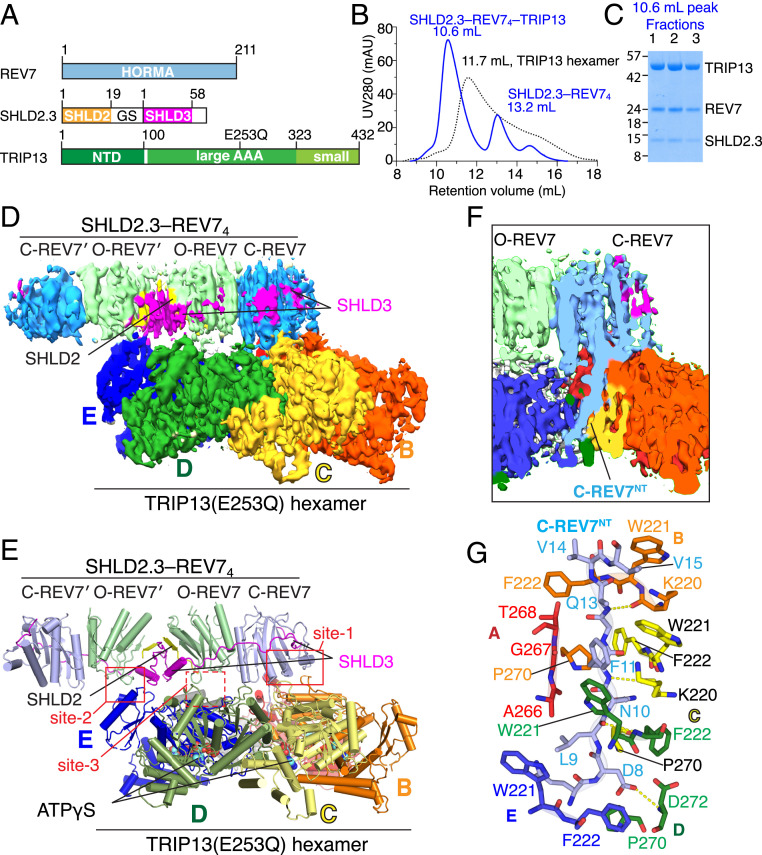
Cryo-EM structure of SHLD2.3–REV7_4_–TRIP13(E253Q) complex. (*A*) Schematic drawing of REV7, SHLD2.3, and TRIP13 (E253Q) proteins involved in complex formation. (*B*) Copurification of the complex formed by TRIP13(E253Q) hexamer and SHLD2.3–REV7_4_ in the presence of ATPγS by size exclusion chromatography. (*C*) SDS-PAGE analysis of fractions from size exclusion chromatography. (*D* and *E*) The overall structure of the SHLD2.3–REV7_4_–TRIP13(E253Q) complex with bound ATPγS shown in electron density (*D*) and ribbon (*E*) representations. The six subunits of TRIP13 are labeled *A* to *F*. (*F* and *G*) Views showing the insertion of the N terminus of C-REV7 into the central pore of the hexameric TRIP13 scaffold in electron density (*F*) and stick (*G*) representations. *G* highlights the interactions between the inserted N terminus (Asp8 to Val14) of C-REV7 and residues from color-coded subunits A to F of TRIP13.

It has been reported that purified AAA^+^ ATPase TRIP13 can dissociate SHLD3–REV7 in vitro in an ATP-dependent manner, as evidenced by the release of REV7 from immobilized GST–SHLD3 complexes (28). We prepared a TRIP13 catalytic mutant (E253Q) that can bind ATP/ATPγS to form a stable hexameric topology but lacks its ATP hydrolysis activity as previously reported (29, 30) (Fig. 4*A*). We next sought to reconstitute the higher-order complex of hexameric TRIP13(E253Q) bound to the SHLD2.3–REV7_4_ complex, which snapshots the initial recognition step of the Shieldin complex by the TRIP13 hexamer. The incubation of purified the SHLD2.3–REV7_4_ and TRIP13(E253Q) hexamer with ATPγS generates a peak eluted at 10.6 mL on an S200 gel filtration column, whereas the TRIP13(E253Q) hexamer alone eluted at 11.7 mL on the same S200 gel filtration column (Fig. 4*B*), indicative of the formation of the SHLD2.3–REV7_4_–TRIP13(E253Q) complex. Sodium dodecyl sulfate–polyacrylamide gel electrophoresis (SDS-PAGE) analysis further confirmed that the TRIP13(E253Q) eluted with REV7 and SHLD2.3 in a 6:4:2 stoichiometry (Fig. 4*C*), consistent with the expected complex composition.

### Cryo-EM Structure of TRIP13 Bound to the SHLD2.3–REV7_4_ Complex.

We next determined the cryo-EM structure of the SHLD2.3–REV7_4_–TRIP13(E253Q) complex with ATPγS (hereafter designated SHLD2.3–REV7_4_–TRIP13 complex; cryo-EM workflow shown in *SI Appendix*, Fig. S5 and cryo-EM statistics in *SI Appendix*, Table S2). Refinement of the electron microscopy dataset yielded an initial consensus reconstruction extended to an average resolution of 3.6 Å (*SI Appendix*, Fig. S5 *B* and *E*), in which clear density was observed for the TRIP13 hexamer, but less so for the SHLD2.3–REV7_4_ component of the complex. The map showed the overall structure of the hexameric ring of TRIP13(E253Q) (*SI Appendix*, Fig. S6 *A* and *B*), that is very similar to that of the TRIP13–p31^comet^-substrate complex (29). Within the hexameric ring of TRIP13, the topology of A to E monomers consists of an N-terminal domain (henceforth denoted NTD), followed by a large AAA^+^ domain and a C-terminal small AAA^+^ domain, that are classical in the AAA^+^ ATPase superfamily. Monomers A to E are bound with ATPγS to form a compact right-handed spiral topology (monomer C with bound ATPγS shown in *SI Appendix*, Fig. S6*C*). By contrast, monomer F is in the apo-state lacking bound ATPγS, leading to the separation of its large and small AAA^+^ domains (*SI Appendix*, Fig. S6*D*), thereby creating a seam in the hexameric ring of TRIP13. The electron density of the NTD of monomer F is not visible, implying the dynamic feature of monomer F in the hexameric ring.

To improve the density for the SHLD2.3–REV7_4_ component, we then used masked local refinement to obtain a focused map at an average resolution of 3.8 Å (*SI Appendix*, Fig. S5*C*). With this map, the secondary structural elements and certain larger side chains of the REV7 tetramer become discernible so that we can dock the crystal structure of the SHLD2.3–REV7_4_ into the significant cryo-EM density feature above the convex face of the TRIP13 hexamer, while most of the REV7 connecting loops, as well as a large portion of SHLD2.3 fragments were still untraceable in the density (*SI Appendix*, Fig. S5*C*). We then used the docked model to combine the consensus and focused maps, so as to obtain a composite map (*SI Appendix*, Fig. S5*D*) for refining the overall structure of the SHLD2.3–REV7_4_–TRIP13 complex. The final structure of such a complex is shown in an electron density view in Fig. 4*D* and its ribbon counterpart in Fig. 4*E*. This overall structure displays a cannon-like architecture,with the SHLD2.3–REV7_4_ constituting a gun barrel fixed on the convex face of the hexameric TRIP13 gun platform. The topology of the SHLD2.3–REV7_4_ component is essentially the same in the crystal structure in the absence of TRIP13 (Fig. 2 *E* and *F*) and the cryo-EM structure with bound TRIP13 (Fig. 4 *D* and *E*). Some examples of electron density tracing between SHLD2.3–REV7_4_ and TRIP13 components spanning interfacial regions in the complex are shown in *SI Appendix*, Fig. S7 *A–D*.

We also generated the SHLD2.3–REV7_2_–TRIP13(E253Q) with ATPγS complex (*SI Appendix*, Fig. S8 *A* and *B*) and collected a cryo-EM dataset on this complex that yielded a consensus map that extended to an average resolution of 3.7 Å (*SI Appendix*, Fig. S8*C*). We observed clear density for the TRIP13 hexamer, but marginal density was observed for the SHLD2.3–REV7 dimer component of the complex, implying that the SHLD2.3–REV7 dimer did not form a stable complex with TRIP13.

### Capture of C-REV7 N Terminus within the Central Channel of TRIP13.

**Fig. 5.**
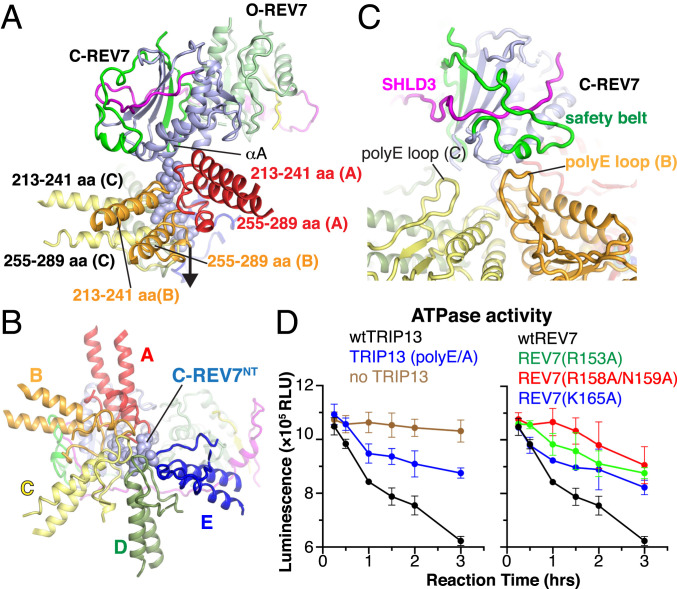
Interactions between REV7 and TRIP13 in the structure of the SHLD2.3–REV7_4_–TRIP13(E253Q) complex. (*A* and *B*) Interaction between the inserted N terminus (Asp8 to Val14) of C-REV7 and pairs of regions containing pore loops (213 to 241 and 255 to 289) from subunits A, B, C, D, and E of TRIP13 aligned in a spiral orientation in the complex. *A* shows a side view while *B* shows a top-down view of the inserted N terminus of C-REV7. (*C*) Interaction between the C-REV7 safety belt and the poly-E loop of TRIP13 in the complex. The main contacts occur between the monomer B poly-E loop and C-REV7 safety belt. The monomer A poly-E loop region is unstructured. (*D*) ATP activity assays of interfacial mutants of TRIP13 and SHLD2L.3–REV7. Data represent three independent experiments with mean ± SD.

Previous cryo-EM structures of substrate-bound AAA^+^ ATPases established that their substrates insert through the conserved central pore, thereby imposing a constriction on the bound substrate polypeptide to eventually force the disassembly of folded topologies (38). We observe that the N terminus (residues 8 to 14, designated REV7^NT^) of C-REV7, which was not visible in the crystal structure in the absence of TRIP13, becomes ordered in our cryo-EM structure of the SHLD2.3–REV7_4_–TRIP13 complex and is threaded through the central pore of TRIP13 (Fig. 4*F*). A detailed view of intermolecular contacts between the precisely inserted Asp8 to Val14 of REV7^NT^ and the color-coded key residues of TRIP13 monomers A to E that constitute the central hydrophobic pore of TRIP13 is shown in Fig. 4*G*. This central pore is lined by pore loops 1 (residues 218 to 224) and 2 (residues 266 to 274) emanating from individual subunits of the ATPase (*SI Appendix*, Fig. S9). In this configuration, the highly conserved TRIP13 Trp-221 and Phe-222 of pore loop 1 of monomers B to E, as well as Pro-270 of pore loop 2 of monomers B to D embrace residues 8 to 14 of REV7^NT^ in a ratcheted manner (Figs. 4*G* and 5 *A* and *B*). By contrast, TRIP13 monomer A merely uses Ala-266, Gly-267, and Thr-268 of its pore loop 2 residues to contact REV7^NT^, while no interactions are observed between TRIP13 monomer F and REV7^NT^ (Fig. 4*G*).

### Interfaces between TRIP13 and SHLD2.3–REV7_4_.

The structure of the SHLD2.3–REV7_4_–TRIP13 complex reveals two direct intermolecular contact surfaces between SHLD2.3–REV7_4_ and TRIP13 components (Fig. 4 *D* and *E*). The primary interface (site-1, Fig. 4*E*) involves contacts between the safety-belt segment of C-REV7 and the conserved and negatively charged loop (110-ENLEEETENII-120; hereafter designated the poly-E loop; *SI Appendix*, Fig. S9) of TRIP13 monomer B (Figs. 5*C* and 4 *D* and *E*). The second contact (site-2; Fig. 4*E*) forms mainly between the loop 88 to 95 of O-REV7′ and the NTD of TRIP13 monomer E (Fig. 4 *D* and *E*). We propose that this second contact could help to reduce the flexibility of SHLD2.3–REV7_4_ upon TRIP13 binding, with the potential limitation that such a contact could reflect the artificial fusion strategy for the generation of SHLD2.3. In addition, we also noted a potential less precisely defined intermolecular contact (site-3; Fig. 4*E*) between loop 88 to 95 of O-REV7 and negatively charged surface contributed by the poly-E loop of TRIP13 monomer E (Fig. 4 *D* and *E*).

Given the above-mentioned limitations of site-2 and site-3, we focused our analysis of the site-1 interface between the C-REV7 safety belt and poly-E loop of TRIP13 monomer B (Fig. 5*C*). We can trace the main chains but not the side chains of the interfacial residues due to the blurry electron density (*SI Appendix*, Fig. S7*B*). To test whether site-1 is essential for TRIP13-induced remodeling of the SHLD2.3–REV7_4_, we replaced 123-EEE-125 of TRIP13 with a triple alanine substitution to generate polyE/A mutant and compared ATPase activity of wild-type and mutated TRIP13 for remodeling of the SHLD2.3–REV7_4_. As shown in Fig. 5*D*, the ATPase activity was reduced in the polyE/A mutant, showing that the negatively charged segment in the poly-E loop is important for TRIP13 function. To test the importance of the REV7 safety-belt sequence, we prepared three alanine substitution mutants of REV7 spanning safety-belt interfacial basic residues (R153A, R158A/N159A, and K165A). These individual sets of REV7 mutations resulted in a clear reduction in the efficacy of the ATPase activity of wild-type TRIP13 (Fig. 5*D*). These mutagenesis data confirm that TRIP13’s ATPase activity is tightly coupled to engagement of site-1 interactions.

### Cryo-EM Analysis of TRIP13(E253Q) Bound to the SHLD2L.3-REV7 Dimer Complex.

**Fig. 6.**
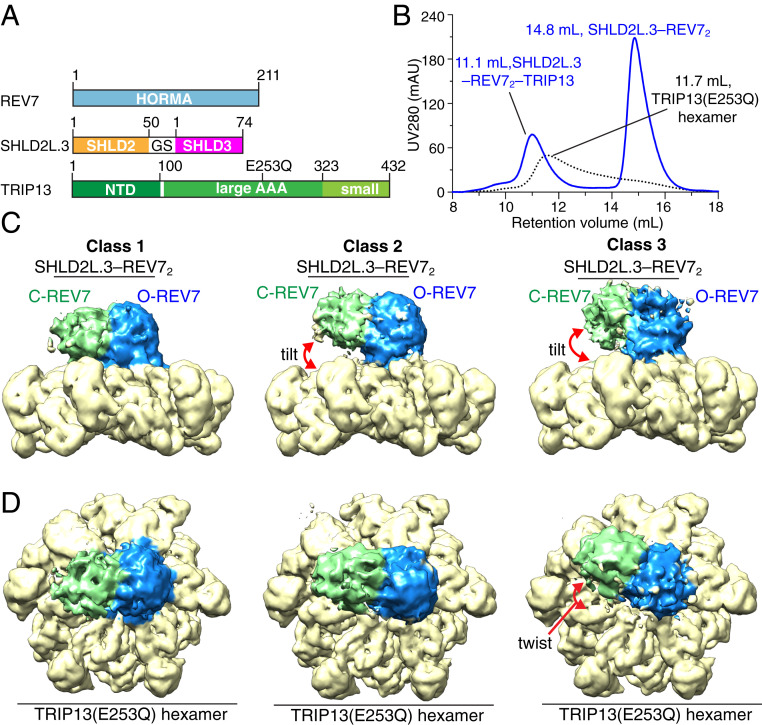
Cryo-EM structure of SHLD2L.3–REV7_2_–TRIP13(E253Q) complex. (*A*) Schematic drawing of REV7, SHLD2L.3, and TRIP13 (E253Q) proteins involved in complex formation. Note that SHLDL2.3 contains a longer version (residues 1 to 50) of SHLD2 compared with SHLD2.3 (residues 1 to 19 of SHLD2). (*B*) Copurification of the complex formed by TRIP13(E253Q) hexamer and SHLD2L.3–REV7_2_ in the presence of ATPγS by size exclusion chromatography. (*C* and *D*) The overall structure of the SHLD2L.3–REV7_2_–TRIP13(E253Q) complex with bound ATPγS shown in electron density representations. Three different classes of structures of the complex with different twist and tilt between the SHLD2L.3–REV7_2_ and TRIP13 (E253Q) components are shown with side (*C*) and top (*D*) views.

The β1 strand of SHLD2 (residues 1 to 19) is sandwiched by the β6 strand of O-REV7 and the N-terminal β1 strand of SHLD3 in our crystal structure of the fused SHLD2.3–REV7_4_ complex (Fig. 2 *G* and *H* and *SI Appendix*, Fig. S10*A*). A crystal structure has also been reported recently of the SHLD2(residues 1 to 52)–SHLD3(residues 1 to 64)–REV7 dimer complex (27). In this structure, SHLD2(residues 1 to 52) forms an additional β-hairpin (β2 and β3) to further sandwich the β1 strand of SHLD3 in the ternary complex (*SI Appendix*, Fig. S10*B*). Accordingly, we decided to replace the SHLD2(residues 1 to 19) of SHLD2.3 by its longer counterpart SHLD2L(residues 1 to 50), to generate fused SHLD2L.3 containing SHLD2L(residues 1 to 50) and a longer SHLD3(residues 1 to 74) linked components (Fig. 6*A*). We observed that such a fused SHLD2L.3–REV7 complex yielded one single peak on a Hitrap Q column and subsequently eluted at 14.8 mL on an S200 gel filtration column (*SI Appendix*, Fig. S10*C*), indicative of the formation of a stable SHLD2L.3–REV7 dimer complex (hereafter labeled SHLD2L.3–REV7_2_). It should be noted that we did not observe the formation of the SHLD2L.3–REV7_4_ complex during purification.

We then reconstituted the SHLD2L.3–REV7_2_–TRIP13(E253Q) complex (Fig. 6*B*) for cryo-EM analysis, with the cryo-EM data work flow shown in *SI Appendix*, Fig. S11 *A* and *B*. Three-dimensional (3D) classification indicated that the component of SHLD2L.3–REV7_2_ exhibited substantial conformational flexibility, with its position tilting or twisting by as much as 30° relative to the TRIP13 hexameric ring (Fig. 6 *C* and *D* and *SI Appendix*, Fig. S11*B*). We did not observe such flexibility in the structure of the SHLD2.3–REV7_4_–TRIP13 complex, perhaps due to its additional site-2 interface that packs with and stabilizes the interaction with the TRIP13 hexamer (Fig. 4 *D* and *E*). Refinement of the 3D classes yielded three consensus maps that extended to average resolutions of 3.9 to 4.1 Å (*SI Appendix*, Fig. S11*B*). We noticed that the SHLD2L.3–REV7_2_ component in the class 1 SHLD2L.3–REV7_2_–TRIP13 complex (Fig. 6 *C* and *D*) shares a similar conformation with the SHLD2.3–REV7_4_ component in the SHLD2.3–REV7_4_–TRIP13 complex (Fig. 4 *D* and *E*). We refrained from building models of classes 1 to 3 of the SHLD2L.3–REV7_2_–TRIP13 complex due to difficulties of peptide tracing into the low-resolution observed densities (*SI Appendix*, Fig. S11*B*).

## Discussion

### Drugability of Site-S on the Rev7 β-Sheet Scaffold.

Given that interactions involving the safety-belt segment of the SHLD3_46–74_–REV7 monomer complex have been highlighted in a recent study of its structure complemented with mutational studies on key intermolecular contacts (26), we do not address related results observed in our study of the SHLD3_35–54_–REV7 monomer complex (Fig. 1). We instead focus on a discussion of the potential of targeting site-S (*SI Appendix*, Fig. S3*A*) as a therapeutic intervention strategy between REV7 and its binding partners (39–41). In contrast to the safety-belt region (*SI Appendix*, Fig. S3*B*) that binds multiple partners (34, 35, 42, 43), site-S, which overlays with the REV1-binding site (*SI Appendix*, Fig. S3*D*) has sufficient complexity, making it ideal for small molecule drug design toward attempts to disrupt the Shieldin/NEHJ pathway. In addition, the site-S interface (*SI Appendix*, Fig. S3*A*) shares one pocket with the adjacent REV1-binding site (42, 44, 45) (*SI Appendix*, Fig. S3*D*) and another adjacent one with a pocket identified by the PockDrug server (*SI Appendix*, Fig. S3*C*), implying the potential for druggability of site-S. Since disrupting of the REV7–REV1 interaction can inhibit mutagenic translesion synthesis (TLS) and reduce chemoresistance (41), such a two-component pocket scaffold (adjacent site-S and REV1-binding site) spanning the REV7 surface could represent an attractive future avenue for improving DNA-damaging chemotherapeutics by simultaneously inhibiting error-prone NHEJ and TLS.

### Impact of Fused SHLD2–SHLD3 Constructs on REV7 Oligomeric Alignment.

In the current study, we report on several structures of fused SHLD2–SHLD3 bound to REV7 in the absence and presence of TRIP13. In one set that includes the X-ray structure of SHLD2.3–REV7_4_ (Fig. 2 *E* and *F*) and the cryo-EM structure of SHLD2.3–REV7_4_–TRIP13 complex (Fig. 4 *D* and *E*), each contain a pair of C-REV7–O-REV7 conformational heterodimers to generate a tetrameric alignment. On the other hand, the cryo-EM structure of the SHLD2L.3–REV7_2_–TRIP13 complex contains only a single C-REV7–O-REV7 conformational heterodimer (Fig. 6 *C* and *D*). We anticipate that REV7 tetramerization reflects the short SHLD2(residues 1 to 19) segment used in the fused SHLD2.3 construct (*SI Appendix*, Fig. S10*A*), which on replacement by the longer SHLD2L(residues 1 to 52) in the SHLD2L.3 construct would disrupt the tetramerization interface due to the presence of additional β2 and β3 segments of SHLD2 (*SI Appendix*, Fig. S10*B*). Thus, we anticipate that the functional complex most likely contains a single C-REV7–O-REV7 conformational heterodimer mediated by bound SHLD2 and SHLD3. However, we cannot rule out the possibility that the tetramer complex could also be physiologically relevant.

### Positioning of the C-REV7–O-REV7 Conformational Heterodimer on the TRIP13 Hexamer on Complex Formation.

We observe that the C-REV7 component of the C-REV7–O-REV7 conformational heterodimer is anchored on the TRIP13 surface primarily as a result of insertion of the C-REV7^NT^ into the central pore of the TRIP13 hexamer channel (Figs. 4 *D* and *E* and 6 *C* and *D*). By contrast the O-REV7 component exhibits flexibility in the SHLD2L.3–REV7_2_–TRIP13 complex as reflected in twist and tilt alignments for this segment (Fig. 6 *C* and *D*). Such flexibility is limited in the structure of the SHLD2.3–REV7_4_–TRIP13 complex, given the additional contacts (labeled site-2) between a second C-REV7–O-REV7 conformational heterodimer and subunit E of TRIP13 in this complex (Fig. 4 *D* and *E*). Stabilization of O-REV7 in the context of a single C-REV7–O-REV7 conformational heterodimer may require the presence of additional factors bound to the complex. In this regard, it should be noted that a recent study established that p31^comet^ promotes homologous recombination by inactivating REV7 through the TRIP13 AAA^+^ ATPase (46). A future structural effort could provide insights into how p31^comet^ promotes TRIP13 to recognize and remodel Shieldin.

### Comparison of C-REV7–O-REV7 Conformational Heterodimer with C-REV7–C-REV7 Homodimer and C-MAD2–O-MAD2 Conformational Heterodimer.

**Fig. 7.**
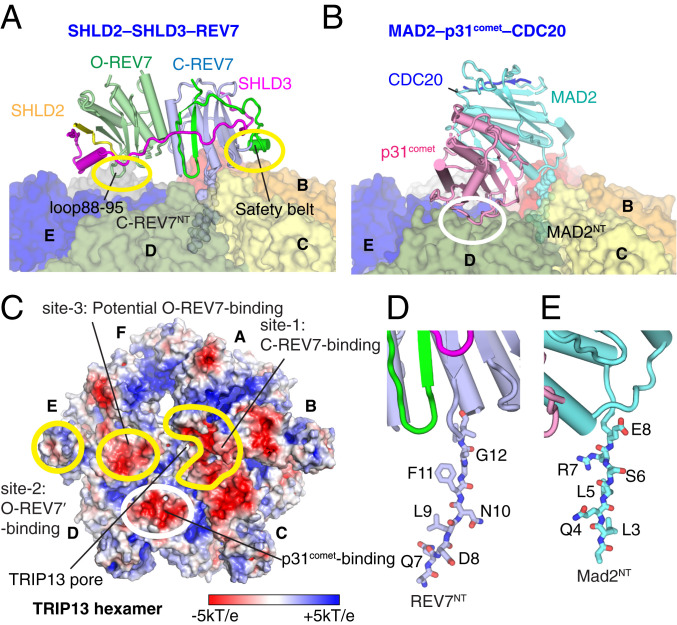
Comparison of structures of SHLD2.3–REV7_4_–TRIP13 and MAD2–p31^comet^–CDC20–TRIP13 complexes. (*A*) Positioning of SHLD2.3-mediated O-REV7–C-REV7 dimer on the surface of TRIP13 following insertion of REV7^NT^ into the central pore of TRIP13 in the SHLD2.3–REV7_4_–TRIP13 structure. (*B*) Positioning of MAD2–p31comet–CDC20 on the surface of TRIP13 following insertion of MAD2^NT^ into the central pore of TRIP13. (*C*) Electrostatic surface representation of the TRIP13 (surface potential at ±5 kT e^−1^). Intermolecular contact patch between REV7 dimer and TRIP13 are highlighted by labeled yellow circles while the intermolecular contact patch between p31comet and TRIP13 is highlighted by a white circle. Monomers *D* and *E* use the consistent acidic surface for p31comet and O-REV7 binding, respectively. The TRIP13 pore is also indicated. The (*D*) REV7^NT^ and (*E*) MAD2^NT^ residues inserted into the TRIP13 pore.

REV7 and its counterparts have multifaceted roles as regulation modules in diverse cellular pathways. Previous structural studies have primarily focused on the binding of monomeric C-REV7 to its partners that contain the consensus REV7-binding motif, and only recently has attention turned to the functional importance of REV7 dimerization in translesion DNA synthesis (37). The latest cryo-EM structure of yeast DNA polymerase ζ reveals a head-to-tail dimer of C-REV7-C-REV7 caused by the two tandem RBMs of REV3 (36) (*SI Appendix*, Fig. S12*A*). By contrast, SHLD3 uses its C-terminal segment to bind C-REV7 and its N-terminal segment to stabilize the O-REV7 and thus result in a conformationally different C-REV7–O-REV7 dimer (29) (*SI Appendix*, Fig. S12*B*). Interestingly, both C-REV7–C-REV7 in the DNA polymerase ζ complex and C-REV7–O-REV7 dimers in the Shieldin complex can be remodeled by TRIP13 (28), supporting our findings that the safety-belt segment of C-REV7 common to both complexes is sufficient for TRIP13 interaction. As an example, the interaction between the C-REV7 safety belt and subunit C of TRIP13 hexamer is shown in Fig. 7*A*.

Structural alignment of the C-REV7–O-REV7 dimer with C-MAD2–O-MAD2 dimer (Protein Data Bank [PDB]: 2V64; *SI Appendix*, Fig. S12*C*) (47), the analog of REV7 functioning in the cell cycle checkpoint, reveals a highly conserved overall architecture (rmsd = 1.487 Å) (*SI Appendix*, Fig. S12*D*). However, the MAD2 homodimer cannot be remodeled by TRIP13. Instead, MAD2 requires its partner p31^comet^ to form a C-MAD2–p31^comet^ heterodimer for recruitment to TRIP13. This is very likely due to the obvious sequence differences between REV7 and MAD2 in their safety-belt regions (*SI Appendix*, Fig. S12*E*). This is supported by the cryo-EM structure of the TRIP13–p31^comet^–MAD2 complex (referred to as TRIP13–p31-substrate complex) showing that the safety-belt element of C-MAD2 forms no direct contacts with TRIP13 (Fig. 7*B*) (29).

### Structural Diversity of C-REV7–O-REV7-Substrate and MAD2–p31-Substrate Recruitment by TRIP13 Hexamer.

The cryo-EM structure of MAD2–p31-substrate–TRIP13 complex (Fig. 7*B*) shows a direct interaction between p31^comet^ and a conserved and negatively charged surface on monomer D (white circle in Fig. 7*C*), mediating the recruitment of the p31-substrate by TRIP13. By contrast, the corresponding surface in the cryo-EM structure of the C-REV7–O-REV7-substrate–TRIP13 complex (Fig. 7*A*) resides on monomer E rather than monomer D (site-3; yellow circle in Fig. 7*C*), thereby providing a potential binding site for the O-REV7–TRIP13 interaction. These data indicate that TRIP13 utilizes different interfaces for substrate recruitment.

### Relative Positioning of Inserted REV7^NT^ and MAD2^NT^ into the TRIP13 Pore.

Most of substrate–pore interactions in AAA^+^ ATPase complexes involve backbone hydrogen bonding and steric contacts to comply with the sequence-independent translocation mechanism (38). In the structures of C-REV7–O-REV7-substrate-TRIP13 and MAD2–p31-substrate-TRIP13 complexes, the REV7^NT^ and MAD2^NT^ residues insert with different registers into the TRIP13 pore (residues 8 to 14 for REV7^NT^ and residues 2 to 9 for MAD2^NT^) (Fig. 7 *D* and *E*). It has been shown previously that the inserted “5-LSR-7” segment of MAD2^NT^ is well defined in the structure (Fig. 7*E*) and important for TRIP13 remodeling (29). A related “4-LTR-6” segment is shared by REV7^NT^, but such a motif is not visible in our structure of the C-REV7–O-REV7-substrate–TRIP13 complex. Instead, we observe the “8-DLNF-12” segment of REV7^NT^ in the TRIP13 pore (Figs. 4*G* and 7*D*). We propose that MAD2^NT^ is inserting the 5-LSR-7 segment midway into the narrow pore at the center of TRIP13 in the MAD2–p31-substrate–TRIP13 complex (Fig. 7*E*), while the 4-LTR-6 segment is captured by the TRIP13 pore during the initial step of insertion, and subsequently completely threaded through the pore, leading the following residues 8 to 14 inserting into the pore in the C-REV7–O-REV7-substrate–TRIP13 complex (Fig. 7*D*). Of note, since we used the noncatalytic TRIP13 mutant and ATPγS in generation of the REV7-substrate–TRIP13 complex, the full insertion of REV7^NT^ into the pore appears to occur in an ATP-independent manner.

### Proposed Remodeling of Shieldin Mediated by ATP-Driven Translocation of TRIP13 Hexamer.

**Fig. 8.**
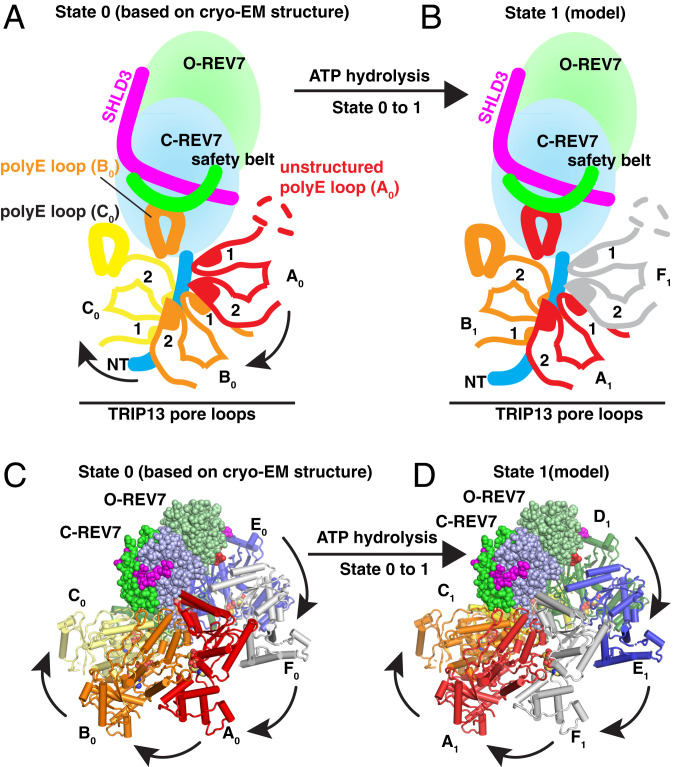
Model of SHLD2.3–REV7 dimer complex remodeling mediated by the ATP-driven translocation of the TRIP13 hexamer. (*A *and *B*)****Schematic of the proposed remodeling mechanism of TRIP13-mediated SHLD2.3–REV7 dimer. The fingers of TRIP13 grip the REV7^NT^ threaded segment tightly and the translocation of TRIP13 monomers draws the thread from REV7 into the channel in stepwise manner. (*C*and* D*) Models of the SHLD2.3–REV7_4_–TRIP13 complexes in basal state 0 and basal state 1 (before and after the first catalytic cycle, see more details in Movie S1). For clarity, only one copy of SHLD2.3–REV7 dimer is shown in a sphere representation. In basal state 0 (*C*), TRIP13 monomers A_0_, B_0_, and C_0_ hold the C-REV7^NT^, while monomer E_0_ contacts O-REV7. As shown in *D*, the first cycle of ATP hydrolysis occurs in monomer E_0_, which transforms from a compact ATP-bound state to the flexible apo-state E_1_; the neighboring seam monomer F_0_ binds one ATP molecule to adopt the ATP-bound F_1_ state. These structural changes cause F_1_ to climb to the top of the AAA^+^ spiral to push an anticlockwise rotation of the SHLD2.3–REV7 dimer, which renders O-REV7 to form new contacts with monomer D_1_.

We define our current cryo-EM structure as the “basal state 0” of the REV7-substrate–TRIP13 complex. To explore how ATP-driven translocation of TRIP13 induces Shieldin remodeling, we modeled the “basal state 1” following the first cycle of catalysis (shown schematically in Fig. 8 *A* and *B* and model in Fig. 8 *C* and *D*). Together with insights from previous reports (29, 38), we propose a model to explain Shieldin remodeling mediated by TRIP13 (Fig. 8 *A*–*D*, Movie S1). In state 0, pore loops 1 and 2 of monomers A_0_, B_0_, and C_0_ hold the C-REV7^NT^ within the central pore (Fig. 8*A*), while the poly-E loop of monomer B_0_ contacts the safety-belt segment of C-REV7 (Fig. 8*A*; site-1 shown in Fig. 4*E*), while monomer E_0_ contacts loop 88 to 95 of O-REV7 (site-3 shown in Fig. 4*E*), with all these interactions contributing to the stabilization of the complex. The first cycle of ATP hydrolysis occurs in monomer E_0_, which transforms from a compact ATP-bound state to a flexible apo-state labeled E_1_, thereby releasing O-REV7 (Movie S1); meanwhile, the neighboring seam monomer F_0_ binds one ATP molecule to adopt an ATP-bound F1 state (Fig. 8 *C* and *D*). The resulting structural changes cause F_1_ to climb to the top of the AAA^+^ spiral to force an anticlockwise rotation of the REV7 substrate relative to TRIP13. In state 1, the pore loops 1 and 2 of monomers F_1_, A_1_, and B_1_ hold the C-REV7^NT^ (Fig. 8*B*), poly-E loop of monomer A_1_ contacts the safety-belt segment of C-REV7 (Fig. 8*B*), while monomer D_1_ contacts loop 88 to 95 of O-REV7 (Fig. 8*D*). The continued stepwise rotations of SHLD2.3–REV7 can eventually cause steric clashes between the REV7 substrate and TRIP13, which likely results in the unwinding and stretching of the polypeptide chain of the αA helix of C-REV7, thereby initiating the unfolding of REV7 and subsequent remodeling of the Shieldin complex.

## Methods

### Protein Expression and Purification.

The codon-optimized DNA sequences were synthesized by Integrated DNA Technologies (IDT). The REV7 sequence was cloned into the MCS1 of the pRSFDuet-1 vector (Novagen) engineered with an N-terminal His_6_-SUMO tag, and the untagged SHLD3s, SHLD3(residues 1 to 58), SHLD2.3, or SHLD2L.3 related sequence was cloned into the MCS2, respectively. The REV7 mutant (R124A) was generated using the QuikChange Site-Directed Mutagenesis Kit. The proteins were expressed in *Escherichia coli* strain BL21 CodonPlus(DE3)-RIL (Stratagene). Bacteria were grown in Luria-Bertani medium at 37 °C to OD_600_ of 0.8 and induced by 0.3 mM isopropyl β-ᴅ-1-thiogalactopyranoside at 18 °C overnight. The protein complex was purified by a HisTrap FF column (GE Healthcare) and the His_6_-SUMO tag was removed by ULP1 protease (laboratory stock) and reloaded on a HisTrap FF column in the buffer (20 mM Tris⋅HCl, pH 8.0, 50 mM NaCl, and 5 mM β-mercaptoethanol). The flow-through was directly loaded on an anion exchange column (HiTrap Q HP, GE Healthcare). The elution was further purified by size exclusion chromatography (Superdex 200 [16/60], GE Healthcare) in the buffer (20 mM Tris⋅HCl [pH 7.5], 150 mM NaCl, and 2 mM dithiothreitol [DTT]). The high-purity eluting fractions were detected by SDS-PAGE and concentrated to around 15 mg/mL. The protein was flash frozen in liquid nitrogen and stored at −80 °C.

### Crystallization and Structure Determination.

The SHLD3s–REV7 complex was crystallized by the hanging drop vapor diffusion method to equilibrate 1.5 μL of SHLD3s–REV7 solution (about 10 mg/mL) with 1.5 μL of reservoir solution (0.2 M ammonium acetate, 0.1 M Na citrate pH 5.6, 30% PEG4000) at 20 °C. The SHLD2.3–REV7_4_ complex was crystallized by the hanging drop vapor diffusion method to equilibrate 1.5 μL of SHLD2.3–REV7 solution (about 4 mg/mL) with 1.5 μL of reservoir solution (0.1 M Na acetate pH 5.6, 1.5 M Na formate) at 20 °C. The crystals were harvested into cryoprotectant solution containing 30% glycerol before being flash frozen by liquid nitrogen. X-ray diffraction data were collected on the 24-ID-C and 24-ID-E beamline at the Advanced Photon Source. The data were autoprocessed by the Northeastern Collaborative Access Team Rapid Automated Processing of Data (RAPD) online server.

Both structures were determined by molecular replacement in PHENIX.Phaser (48, 49) using the modified REV7–REV3 complex structure (PDB code 3ABD) as the search model. Iterative rounds of model building and refinement were performed in COOT (50) and PHENIX.refine (51). Structure factors and final coordinates were deposited (PDB code 6WW9 and 6WWA). Data collection and refinement statistics are shown in *SI Appendix*, Table S1.

### Expression and Purification of TRIP13 Complexes.

The codon-optimized TRIP13 DNA sequence was synthesized by IDT and cloned into the pRSFDuet-1 vector (Novagen) engineered with an N-terminal His_6_-SUMO tag. The TRIP13 mutant (E253Q) was generated using QuikChange. The wild-type and mutant proteins were expressed in *E. coli* strain BL21 CodonPlus(DE3)-RIL (Stratagene). As previously reported (29, 30), the TRIP13 hexamer was purified by anion exchange (HiTrap Q HP) and size exclusion (Superdex 200 [16/60]) chromatography in the buffer (20 mM Hepes pH 7.3, 100 mM NaCl, and 1 mM DTT). The high-purity eluting fractions were detected by SDS-PAGE and concentrated to around 5 mg/mL.

### Reconstitution of SHLD2.3–REV7_4_–TRIP13(E253Q) and SHLD2L.3–REV7_2_–TRIP13(E253Q) Complexes for EM Analysis.

The purified SHLD2.3–REV7_4_ proteins were incubated with purified TRIP13(E253Q) at 2:1 molar ratio (accounting for SHLD2.3–REV7_4_ and TRIP13 hexamer) in an assembly reaction buffer (20 mM Hepes pH 7.3, 100 mM NaCl, 5 mM MgCl_2_, 2 mM ATPγS, and 1 mM DTT) for 1 h at 23 °C. The assembly reaction was then purified by size exclusion chromatography (Superdex 200 [16/60]) with a running buffer containing 20 mM Hepes pH 7.3, 300 mM NaCl, 5 mM MgCl_2_, 0.1 mM ATPγS, and 1 mM DTT. The complex eluted in a peak fraction at a concentration of 0.3 mg/mL and was used to prepare cryo-EM grids. The same protocol was applied to reconstitute the SHLD2L.3–REV7_2_–TRIP13(E253Q) complex.

### Cryo-EM Data Collection.

A total of 3.0 µL of 0.3 mg/mL complex samples was applied onto glow-discharged UltrAuFoil 300 mesh R1.2/1.3 grids (Quantifoil). Grids were blotted for 1.5 s at around 100% humidity and 4 °C and plunge frozen in liquid ethane using an FEI Vitrobot Mark IV. Images were collected on a FEI Titan Krios electron microscope operating at 300 kV with a Gatan K3 camera. All data were collected using a set defocus range of −1.0 µm to −2.5 µm with a pixel size of 1.064 Å at the Richard Rifkind Center for Cryo-EM at Memorial Sloan Kettering Cancer Center. Movies were recorded in superresolution mode at an electron dose rate of 20 e^−^/pixel/s with a total exposure time of 3 s, for an accumulated electron dose of 53 e^−^/Å2. Intermediate frames were recorded every 0.075 s for a total number of 40 frames.

### Cryo-EM Image Processing.

Drift correction of the movie frames was performed with MotionCor2 (52). Contrast transfer function parameters were estimated by CTFFIND4 (53). All other steps of image processing were carried out with RELION3 (54). All reported map resolutions are from gold-standard refinement procedures with the Fourier shell correlation (FSC) = 0.143 criterion after postprocessing by applying a soft mask. For data from the SHLD2.3–REV7_4_–TRIP13(E253Q) complex, automated particle selection resulted in 1,212,927 particles from 1,363 images. After two rounds of two-dimensional (2D) classification, a total of 846,918 particles were selected for 3D classification using the TRIP13–p31-substrate model (PDB 6F0X) as reference. Particles corresponding to the best class with the highest-resolution features were selected and subjected to the second round of 3D classification. One of 3D classes showed extra density of SHLD2.3–REV7_4_, and the corresponding 104,023 particles were polished using RELION particle polishing, yielding a consensus electron microscopy map with a resolution of 3.6 Å after 3D autorefinement. The masked local refinement by applying a soft mask around the SHLD2.3–REV7_4_ density provides a focused electron microscopy map with a resolution of 3.8 Å. Local resolution estimations were calculated from two half data maps with RELION3. Further details related to data processing and refinement are summarized in *SI Appendix*, Table S2.

As for data from the SHLD2L.3–REV7_2_–TRIP13(E253Q) complex, automated particle selection resulted in 1,030,830 particles from 2,438 images. After two rounds of 2D classification, a total of 925,535 particles were selected for 3D classification using the TRIP13–p31-substrate model (PDB 6F0X) as reference. A total of 414,774 particles corresponding to the best two classes with the highest-resolution features were selected and subjected to the second round of 3D classification with applying a soft mask around the SHLD2L.3–REV7_2_ density. Three of the 3D classes showed extra density of SHLD2L.3–REV7_2_ and the corresponding 69,882, 61,657, and 43,949 particles were selected, yielding three consensus electron microscopy maps with resolutions of 3.9, 3.9, and 4.1 Å after 3D autorefinement. Local resolution estimations were calculated from two half data maps with RELION3 (54).

### Atomic Model Building and Refinement of Cryo-EM Data.

The consensus and focused maps of the SHLD2.3–REV7_4_–TRIP13(E253Q) complex were aligned and combined with the PHENIX.CombineFoucsedMaps (48) to construct a composite map to 3.6 Å, the resolution of the consensus map, and refinement was carried out at this resolution. To build the model, the crystal structure of the SHLD2.3–REV7_4_ complex (this work) and cryo-EM structure of TRIP13(E253Q) (PDB 6F0X) were docked into the composite map using UCSF Chimera and then manually rebuilt in COOT as needed. All models were refined against the composite maps using Phenix.real_space_refine (55) by applying geometric and secondary structure restraints. All figures were prepared by PyMol (https://pymol.org/2/) or UCSF Chimera (56). The statistics for data collection and model refinement are shown in *SI Appendix*, Table S2.

### SEC-MALS Experiments.

For protein molar mass determination, purified SHLD3s–REV7, SHLD3 (1 to 58)–REV7, and SHLD2.3–REV7 proteins were analyzed using an ÄKTA-MALS system. A mini DAWN TREOS multiangle light scattering detector (Wyatt Technology) and an Optilab T-rEX refractometer (Wyatt Technology) were used in-line with a Superdex200 10/300 gel filtration column (GE Healthcare) preequilibrated in the buffer (20 mM Tris⋅HCl [pH 7.5], 150 mM NaCl, and 2 mM DTT) at a flow rate of 0.2 mL/min. Separation and ultraviolet (UV) detection were performed by ÄKTA Pure System (GE Healthcare), light scattering was monitored by the mini DAWN TREOS system, and concentration was measured by the Optilab TrEX differential refractometer. Molar masses of proteins were calculated using the Astra 6.1 program (Wyatt Technology) with a dn/dc value (refractive index increment) of 0.185 mL/g. The data were plotted using Prime8 software (GraphPad).

### ATP Activity Assay of TRIP13 in the Presence of SHLD2L.3–REV7 Substrate.

The SHLD2L.3–REV7 dimer constructions containing REV7 mutations as well as TRIP13 with the E113A/E114A/E115A mutation (refers to polyE/A) were generated using QuikChange and confirmed by sequencing. The expression and purification of these mutants are the same as those for the wild-type proteins. A total of 2 μM of wild-type or mutated TRIP13 was incubated with 10 μM SHLD2L.3–REV7 substrates at 37 °C in 100 μL reaction buffer (20 mM Hepes pH 7.3, 100 mM NaCl, 5 mM MgCl_2_, 10 mM ATP, and 1 mM DTT). As for indicated time points, 10 μL of reaction solution was taken out and added into a 384-well flat bottom white polystyrene microplate (Greiner Bio-One), and then mixed with 10 μL Kinase-Glo reagent (Promega) for 10 min at room temperature. The luminescence signals were measured using a TECAN Infinite M1000 reader with default mode (1,000 ms, no reduction). The data were analyzed with Prism 8 software (GraphPad).

## Supplementary Material

Supplementary File

Supplementary File

## Data Availability

The atomic coordinates have been deposited in the Research Collaboratory for Structural Bioinformatics Protein Data Bank with the codes 6WW9 (SHLD3s–REV7 complex), 6WWA (SHLD2.3–REV7_4_ complex), and 7L9P [SHLD2.3–REV7_4_–TRIP13(E253Q) complex]. Cryo-EM density maps have been deposited in the Electron Microscopy Data Bank with accession code EMID-23244 [SHLD2.3–REV7_4_–TRIP13(E253Q) complex].
