# Supplementary material for: Molecular mechanisms of assembly and TRIP13-mediated remodeling of the human Shieldin complex
Source: Proc Natl Acad Sci U S A. 2021 Feb 17;118(8):e2024512118. doi: 10.1073/pnas.2024512118 (PMC7923543; doi:10.1073/pnas.2024512118)
Supplement: Supplementary File [file pnas.2024512118.sapp.pdf]

PNAS
www.pnas.org

Supplementary Information for

Molecular mechanisms of assembly and TRIP13-mediated remodeling of the human Shieldin complex

Wei Xie[1,*], Shengliu Wang[1], Juncheng Wang[1], M. Jason de la Cruz[1], Guotai Xu[2], Maurizio Scaltriti[2], and Dinshaw J. Patel[1,*]

[1] Structural Biology Program
Memorial Sloan Kettering Cancer Center
New York, NY 10065, USA

[2] Human Oncology and Pathogenesis Program
Memorial Sloan Kettering Cancer Center
New York, NY 10065, USA

* Correspondence: W.X. (xiew@mskcc.org) and D.J.P. (pateld@mskcc.org);

This PDF file includes:

SI Figure and Video Captions
Figs. S1 to S12
Tables S1 to S2

SI FIGURE CAPTIONS

Fig. S1: Asymmetric unit of the SHLD3s–REV7 monomer complex and tracing of electron density of bound SHLD3s. (*A*) One asymmetric unit contains two copies of SHLD3s-REV7 monomer complex. A black box highlights the 'safety-belt' segment. *(B)* 2Fo-Fc electron density of SHLD3s at a contour level of 1σ.

Fig. S2. Visualization and intermolecular contacts involving the 'safety-belt' segment of the SHLD3s-REV7 monomer complex. (*A, B*) Two alternate views highlighting the threading of SHLD3 within a channel in the REV7 monomer associated with the 'safety-belt' concept. (*C, D*) Hydrophobic (panel *C*) and hydrogen bonding (panel *D*) interactions involving the 'safety-belt 'segment of the complex. SHLD3 residues Pro-50, Leu-51, and Arg-52 form one β strand, interacting with REV7's β6 and β7 strands (residues Val-150, Ile-172, Leu-173, and Ala-174) by backbone hydrogen bonds, assembling into an antiparallel sheet. In addition, SHLD3 Pro-53 and Pro-57 stack with Trp171 and Tyr63 of REV7 respectively, while the backbone carbonyl oxygens of SHLD3 Arg-55 and Pro-58 form two hydrogen bonds with the hydroxy groups in the side chain of REV7 Tyr-63 and Tyr-37.

Fig. S3. Location of various binding sites on the surface of REV7 monomer. (*A-C*) Location of 'site-S' (panel *A*), REV1-binding site (panel *B*) and 'PockDrug' site (panel *C*) on the β-sheet surface of REV7 monomer. Note that the two-component pocket scaffold (site-S and REV1-binding site) spanning REV7 monomer surface represents an attractive drug design target.

Fig. S4: 2Fo-Fc electron densities related to x-ray structure of SHLD2.3–REV7$_4$ complex at a contour level of 1σ. (*A*) Electron density for overall structure of SHLD2.3–REV7$_4$ complex. (*B*) Close-up view of electron density for SHLD2 in the complex. (*C*)**,** Close-up view of electron density for SHLD3 in the complex.

Fig. S5: Cryo-EM Reconstruction of SHLD2.3–REV7$_4$–TRIP13(E253Q) complex. (*A-D*) Flow chart of image processing of SHLD2.3–REV7$_4$–TRIP13(E253Q) complex. Final 3D reconstructed maps (consensus, focused and composite) colored according to local resolution estimation with RELION3. The focused map greatly improved the density quality in SHLD2.3–REV7$_4$ segment. (*E*) Fourier Shell Correlation (FSC) curve of SHLD2.3–REV7$_4$–TRIP13(E253Q) complex and between two half maps (consensus and focused) that were calculated from two half datasets (FSC=0.143 indicated), and between the composite cryo-EM map and corresponding model (FSC=0.5 indicated).

Fig. S6. Structure of the TRIP13(E253Q) hexamer in the SHLD2.3–REV7$_4$–TRIP13(E253Q) complex. (*A, B*) Hexameric ring topology of TRIP13(E253Q) in the SHLD2.3–REV7$_4$–TRIP13(E253Q) complex shown in a ribbon (panel A) and electrostatic surface representation (panel B). ATPγS in a space-filling representation is bound to subunits A, B, C, D and E, but not F. (*C, D*) The closed folding topology of ATPγS-bound TRIP13(E253Q) subunit C and the open folding topology of ATPγS-free TRIP13(E253Q) subunit F.

Fig. S7. Cryo-EM density for interacting segments in the SHLD2.3–REV7$_4$–TRIP13(E253Q) complex. (*A*) Density for the N-terminal C-REV7 segment that inserts into the central pore of the hexameric TRIP13 ring topology. (*B*) Density for the polyE segments of TRIP13 subunits B and C. (*C*) Interaction between the αA helix of C-REV7 and finger helix 213-241 of TRIP13 A-subunit. (*D*) Interaction between loop 88-95 of O-REV7 and residues 104-127 and 230-240 of TRIP13 E-subunit.

Fig. S8: Cryo-EM study of SHLD2.3–REV7$_2$–TRIP13(E253Q) complex. (*A*) Co-purification of the complex formed by TRIP13(E253Q) hexamer and SHLD2.3–REV7$_2$ in the presence of ATPγS by size-exclusion chromatography. (*B*) SDS-PAGE analysis of fractions from size-exclusion chromatography. (*C*) The overall structure of the SHLD2.3–REV7$_2$–TRIP13(E253Q) complex with bound ATPγS shown in electron density.

Fig. S9. Key interactions between C-REV7 and TRIP13 subunit B in the structure of the SHLD2.3–REV7$_4$–TRIP13 complex. Interaction between the inserted N-terminus (Asp8 to Val14) of C-REV7 and pore loops 1 and 2 from subunit B of TRIP13 and interaction between the C-REV7 'safety-belt' and the poly-E loop of TRIP13 subunit B in the complex.

Fig. S10. Rationale for replacing SHLD2.3 by SHLD2L.3 for generating a stable dimeric REV7 complex. (*A, B*) Alignment of SHLD3 β1–SHLD2 β1–O-REV7 β6 to form a β-sheet in the SHLD2.3–REV7$_4$ (panel A, this work) and SHLD2–SHLD3–REV7 dimer (panel B, PDB 6KTO) complexes. (*C*) Sizing column elution pattern for SHLD2L.3–REV7$_2$ complex.

Fig. S11. Cryo-EM Reconstruction of SHLD2L.3–REV7$_2$–TRIP13(E253Q) complex. The numbers of particles are shown above each of the three 3D classes. 3D reconstructed maps are colored according to local resolution estimation with RELION-3. 3D classification of the particles indicates conformational flexibility of the SHLD2L.3–REV7$_2$ in the complex as also shown in Fig. 6 C and D.

Fig. S12. Structural and sequential comparison of REV7 and its paralog MAD2. (*A*) Structure of C-REV7–C-REV7 dimer mediated by REV3, as revealed by the yeast DNA polymerase ζ cryo-EM structure (PDB 6V93). (*B, C, D)* Structural comparison and superposition of REV7 conformational dimer (this work, panel B) and MAD2 conformational dimer (PDB 2V64, panel C). The two structures share a canonical dimeric architecture, with R.M.S.D=1.487 Å (panel D). *(E)* Secondary structures and structure-based sequence alignment of human REV7 and MAD2. The orange boxes highlight the loop 88-95 (site-3) and safety belt (site-4) regions that contact with TRIP13 but are not conserved between REV7 and MAD2.

Supplementary Video 1. Movie of SHLD2.3–REV7 dimer complex remodeling mediated by the ATP-driven translocation of TRIP13 hexamer. The TRIP13 hexamer is shown in ribbon representation while the SHLD2.3–REV7 dimer and ATPγS are show in space-filling representation.

Xie et al., Figure S1

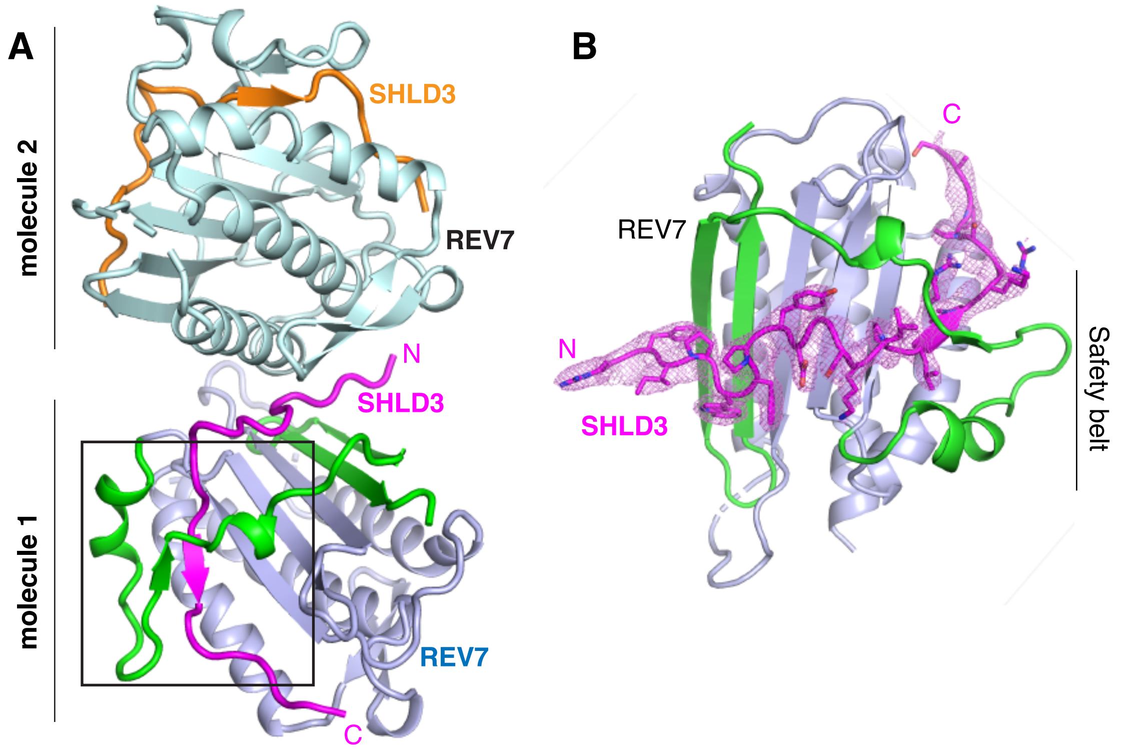

Xie et al., Figure S2

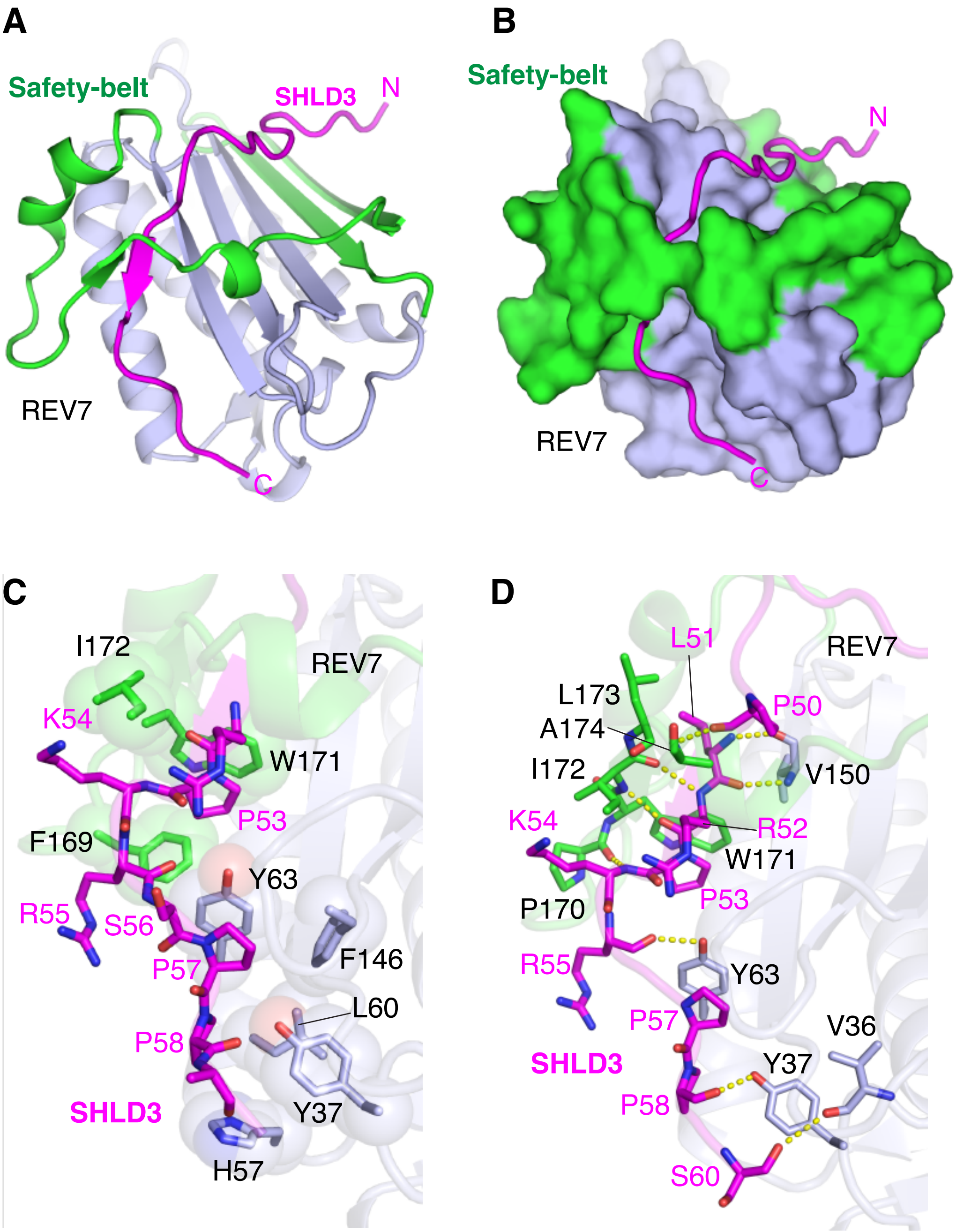

Xie et al., Figure S3

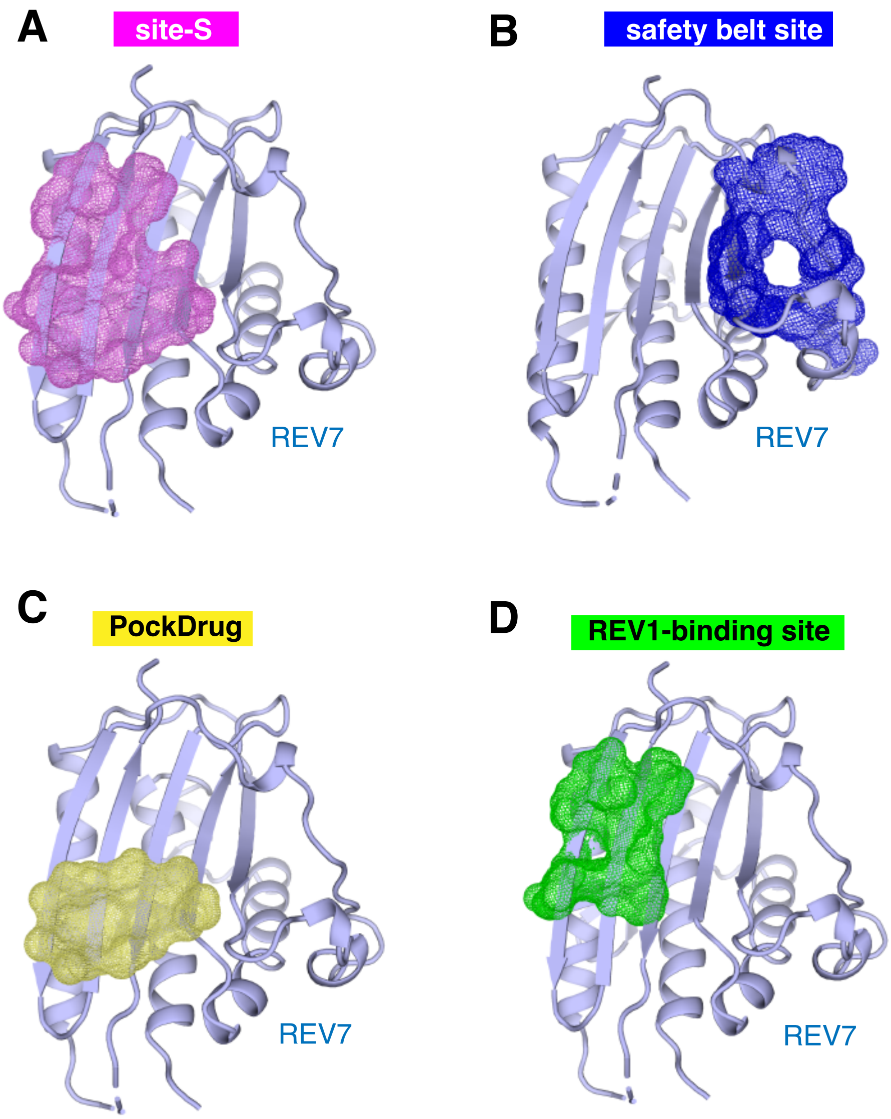

Xie et al., Figure S4

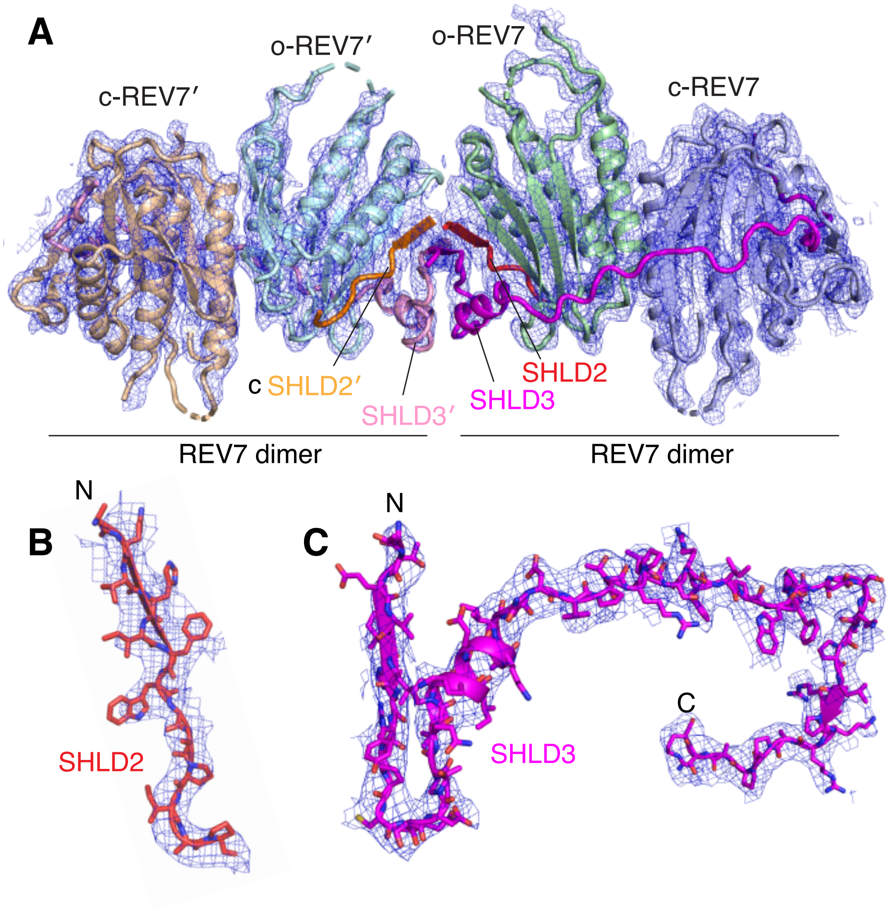

Xie et al., Figure S5

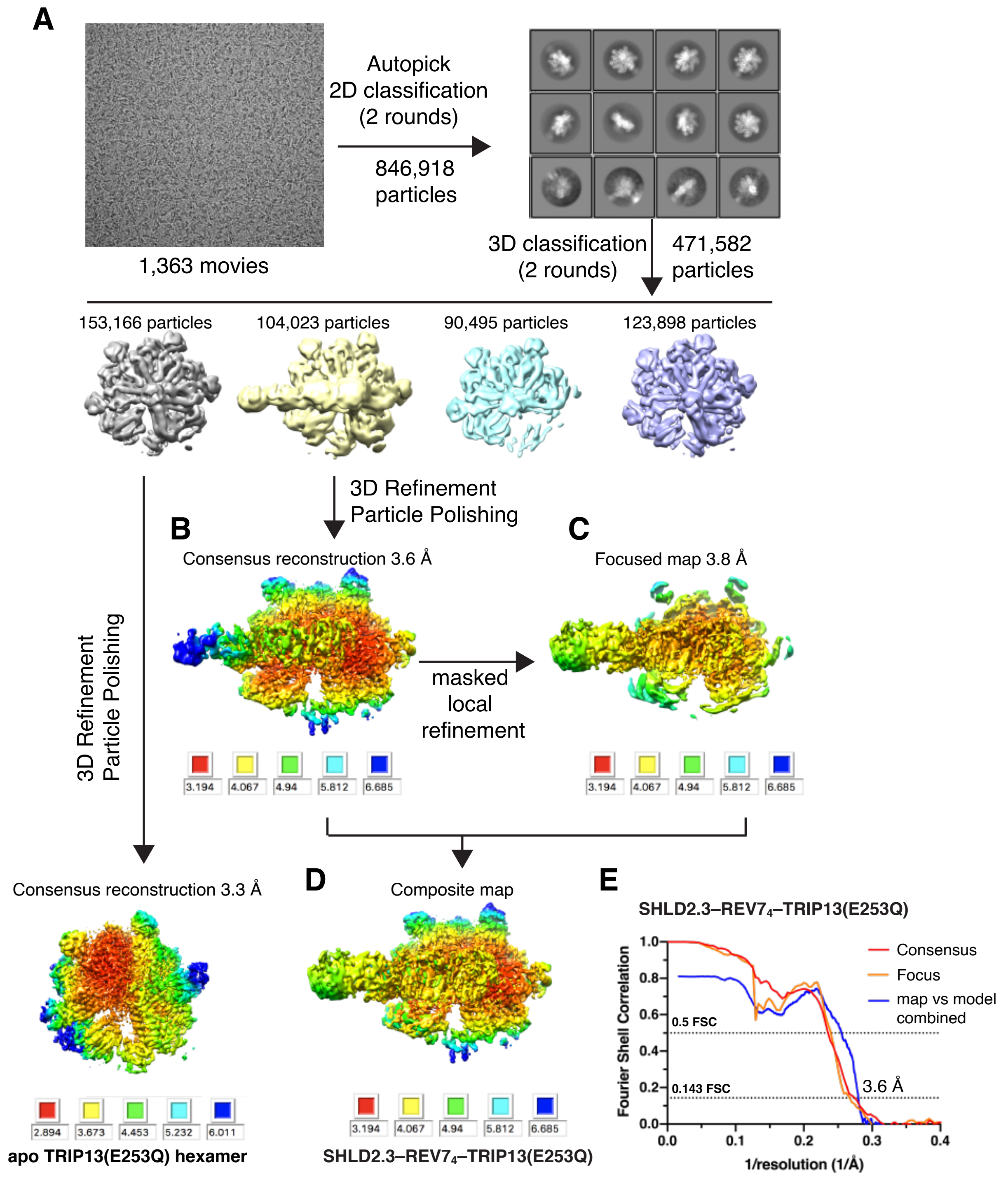

Xie et al., Figure S6

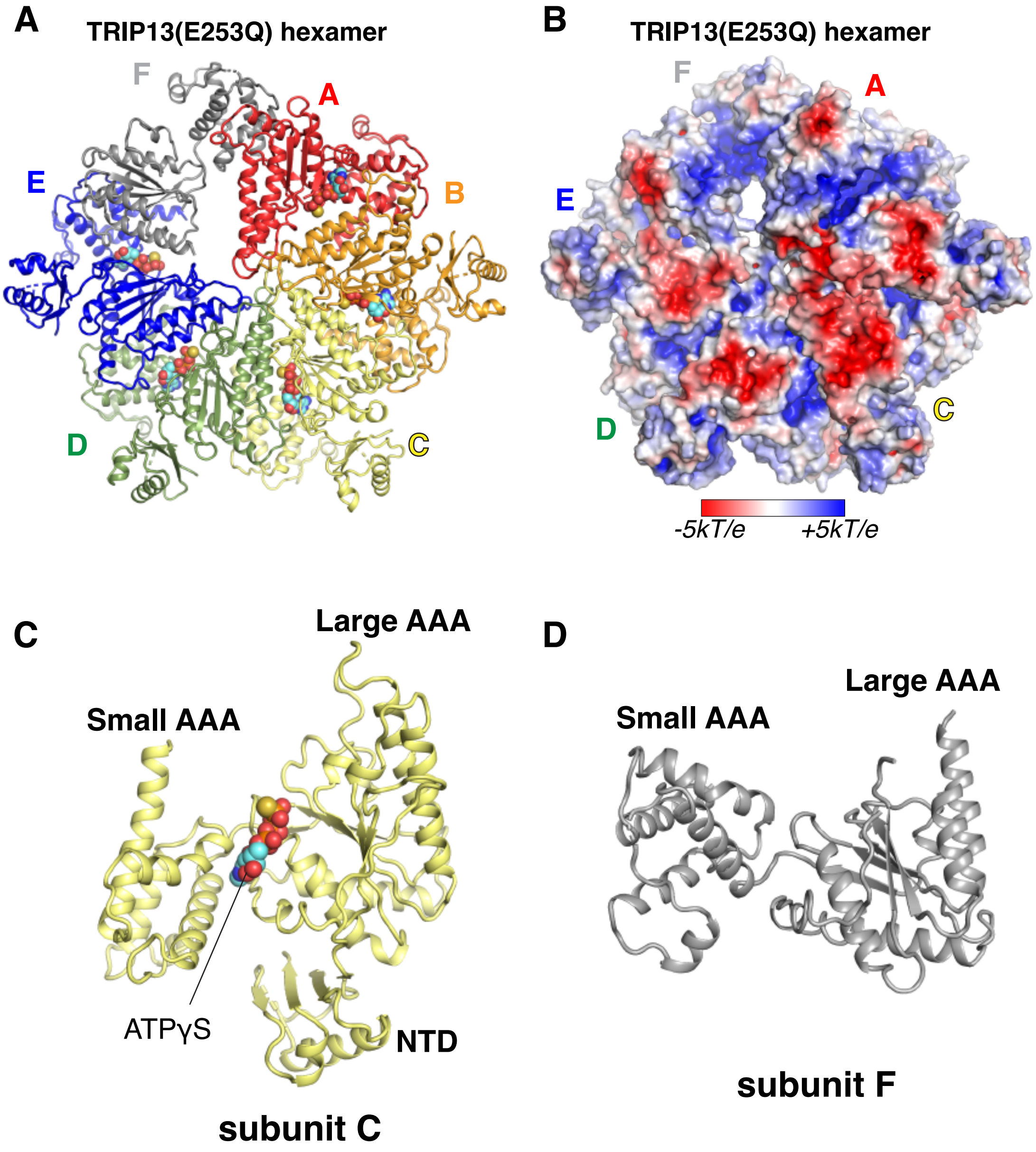

Xie et al., Figure S7

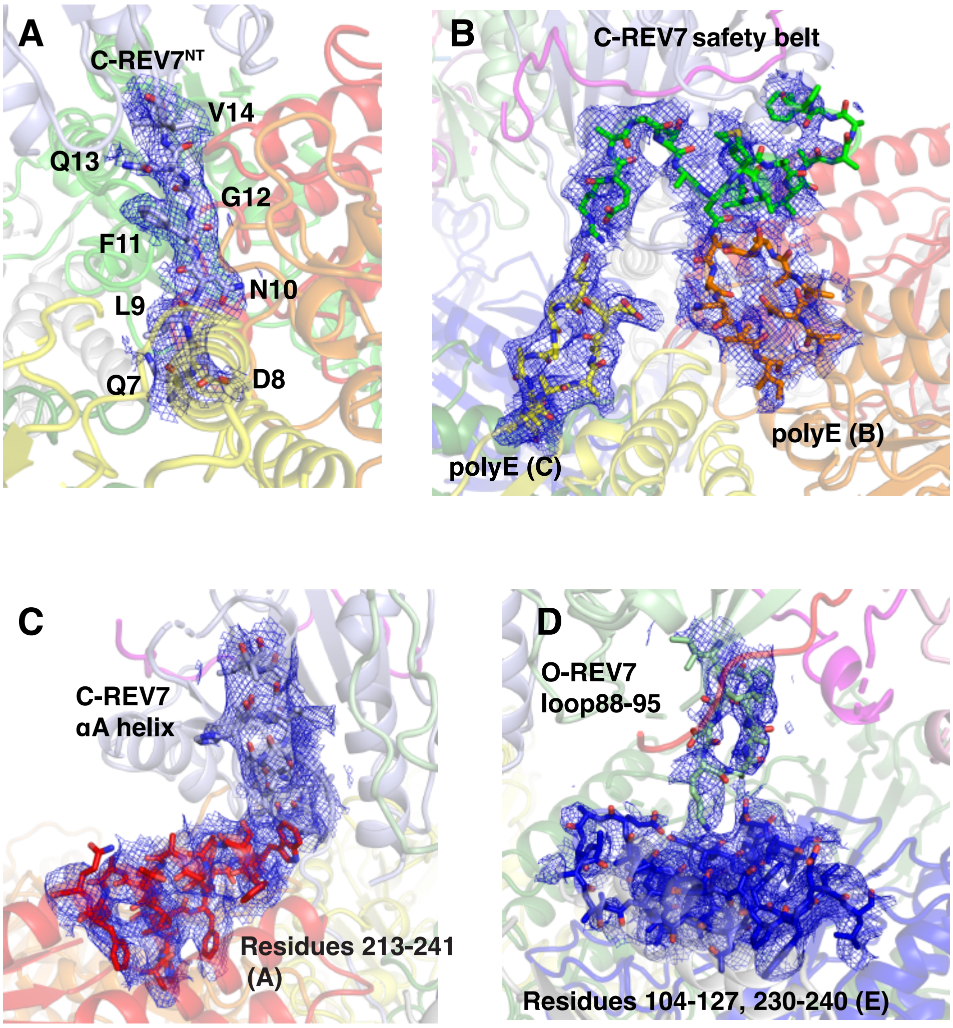

Xie et al., Figure S8

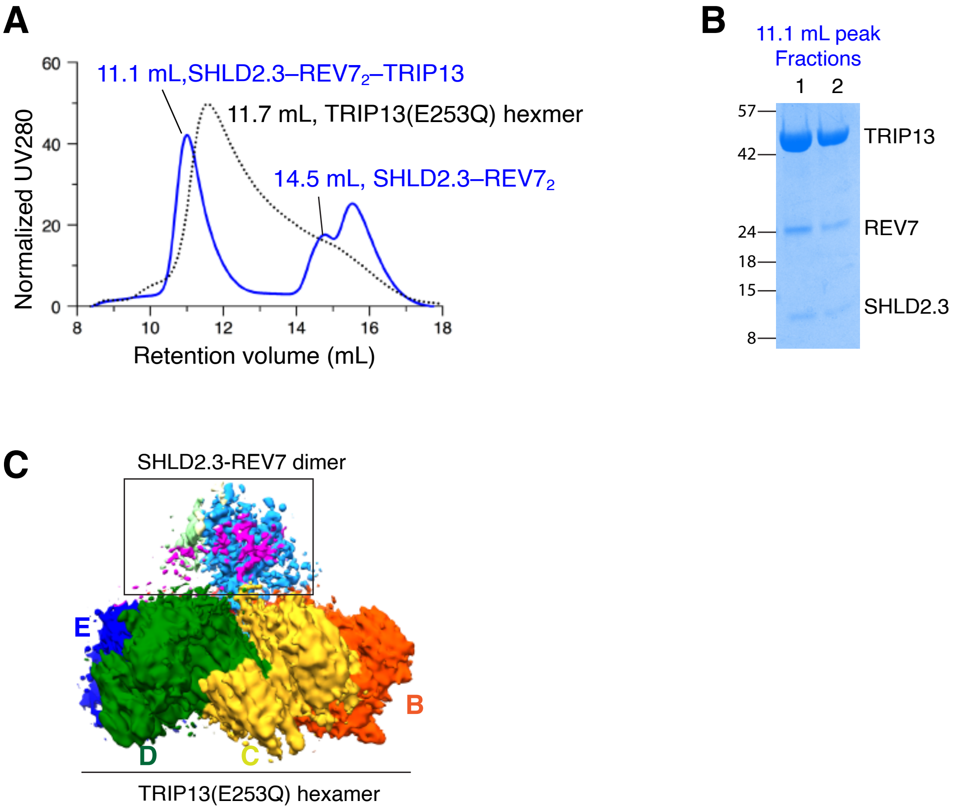

Xie et al., Figure S9

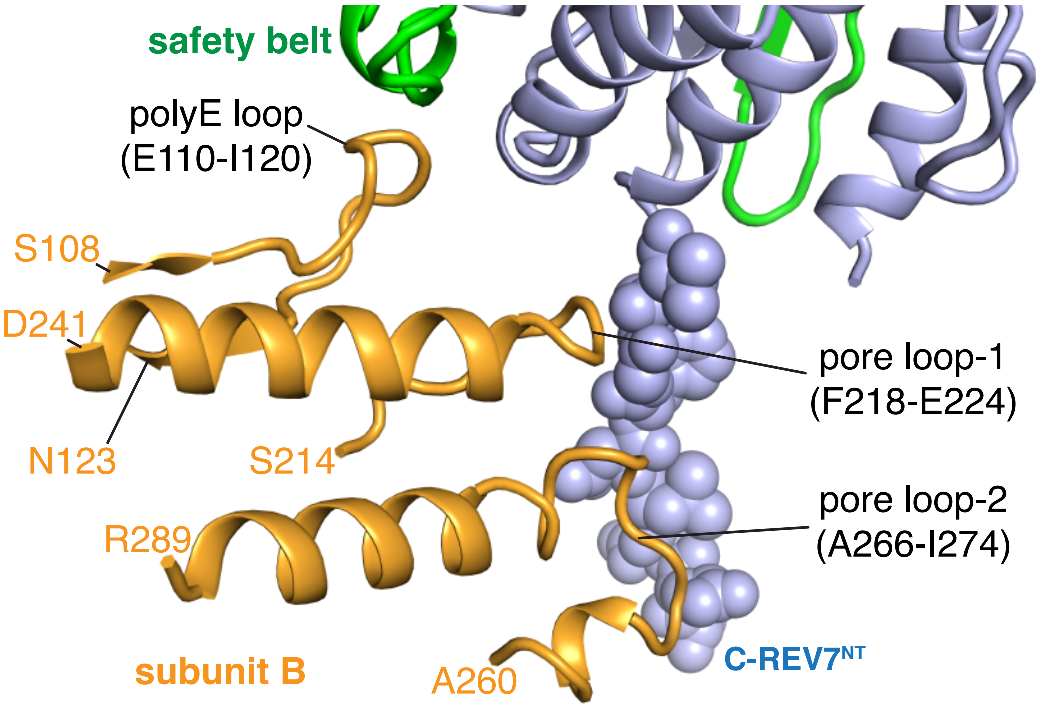

Xie et al., Figure S10

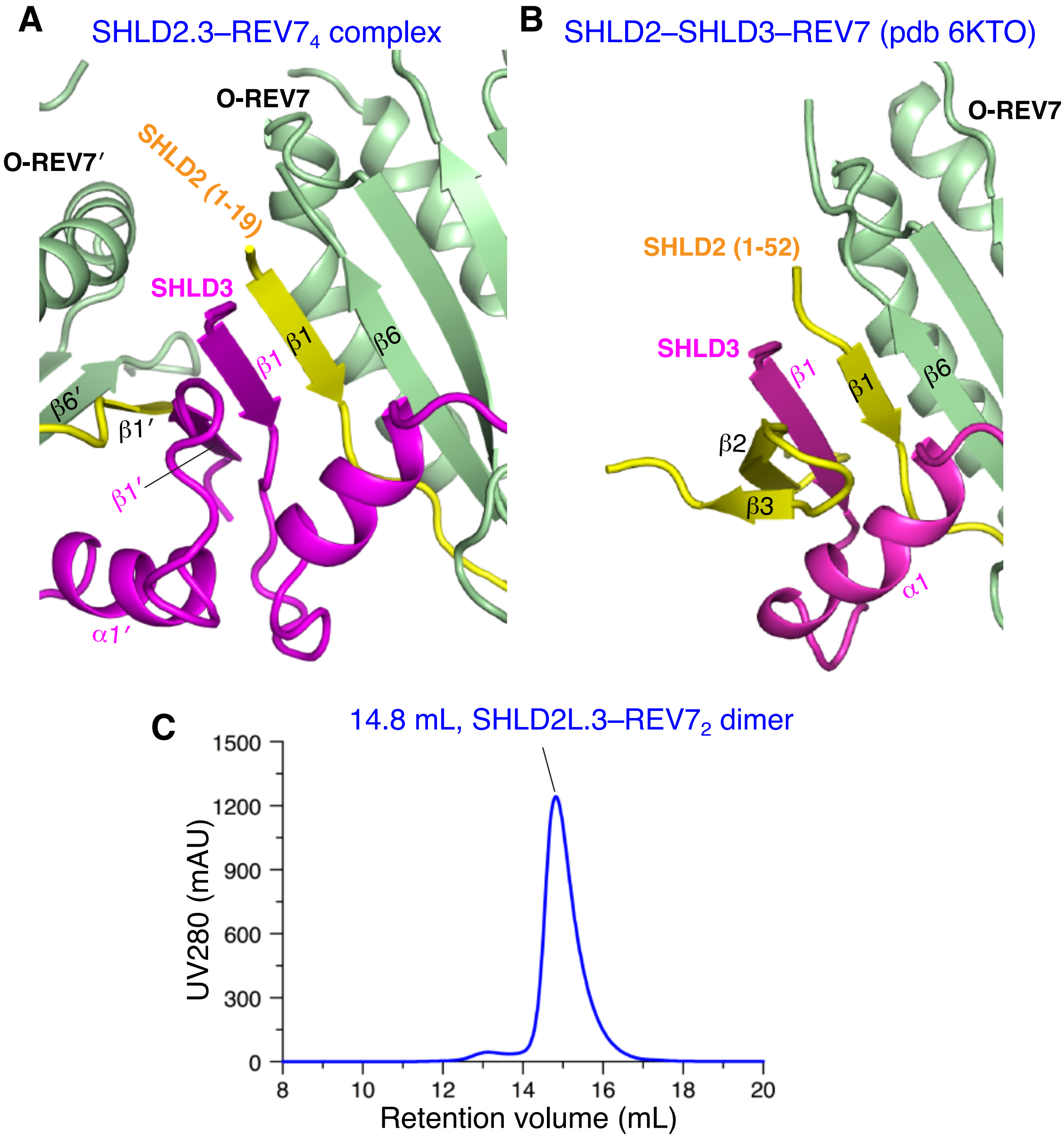

Xie et al., Figure S11

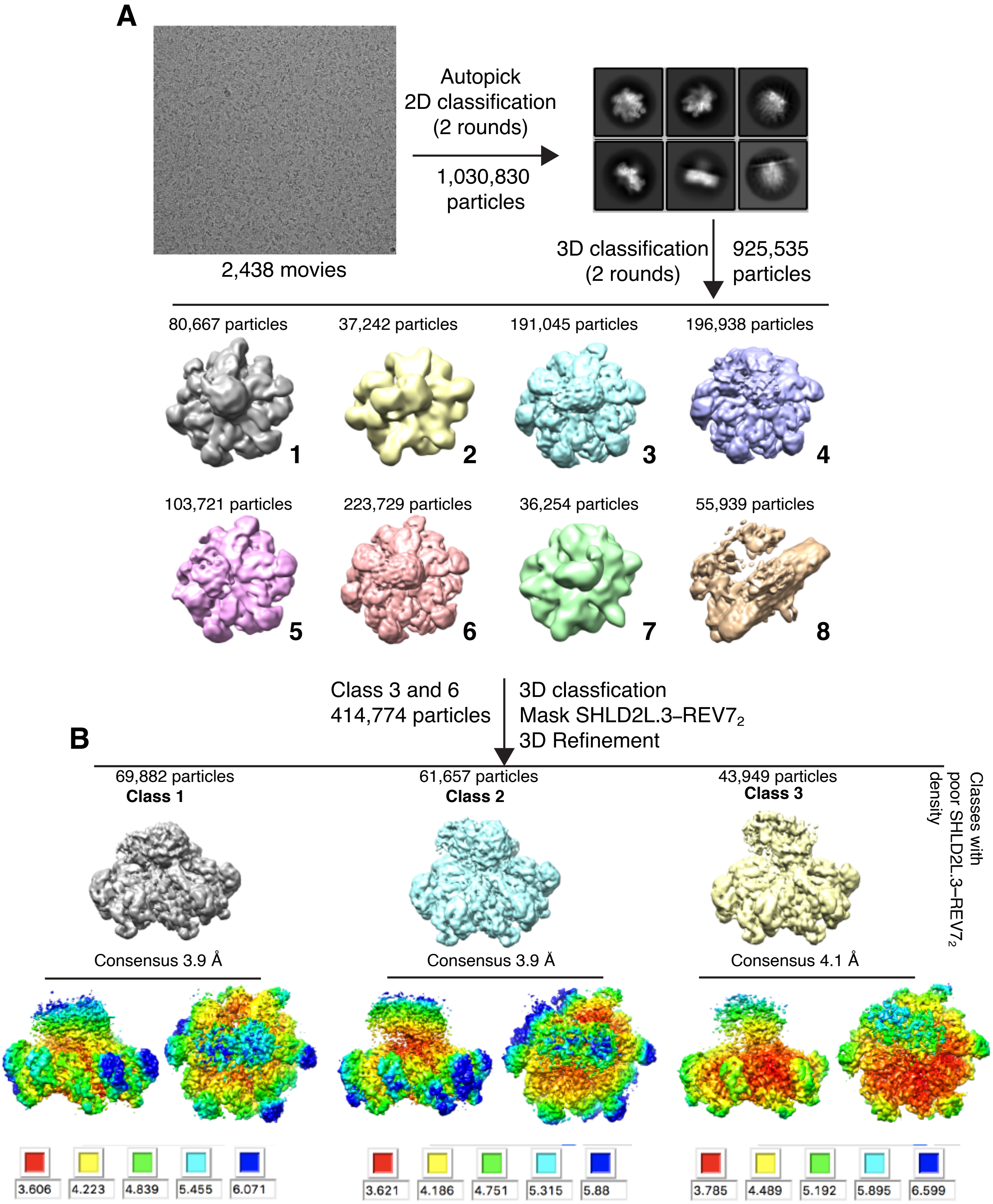

Xie et al., Figure S12

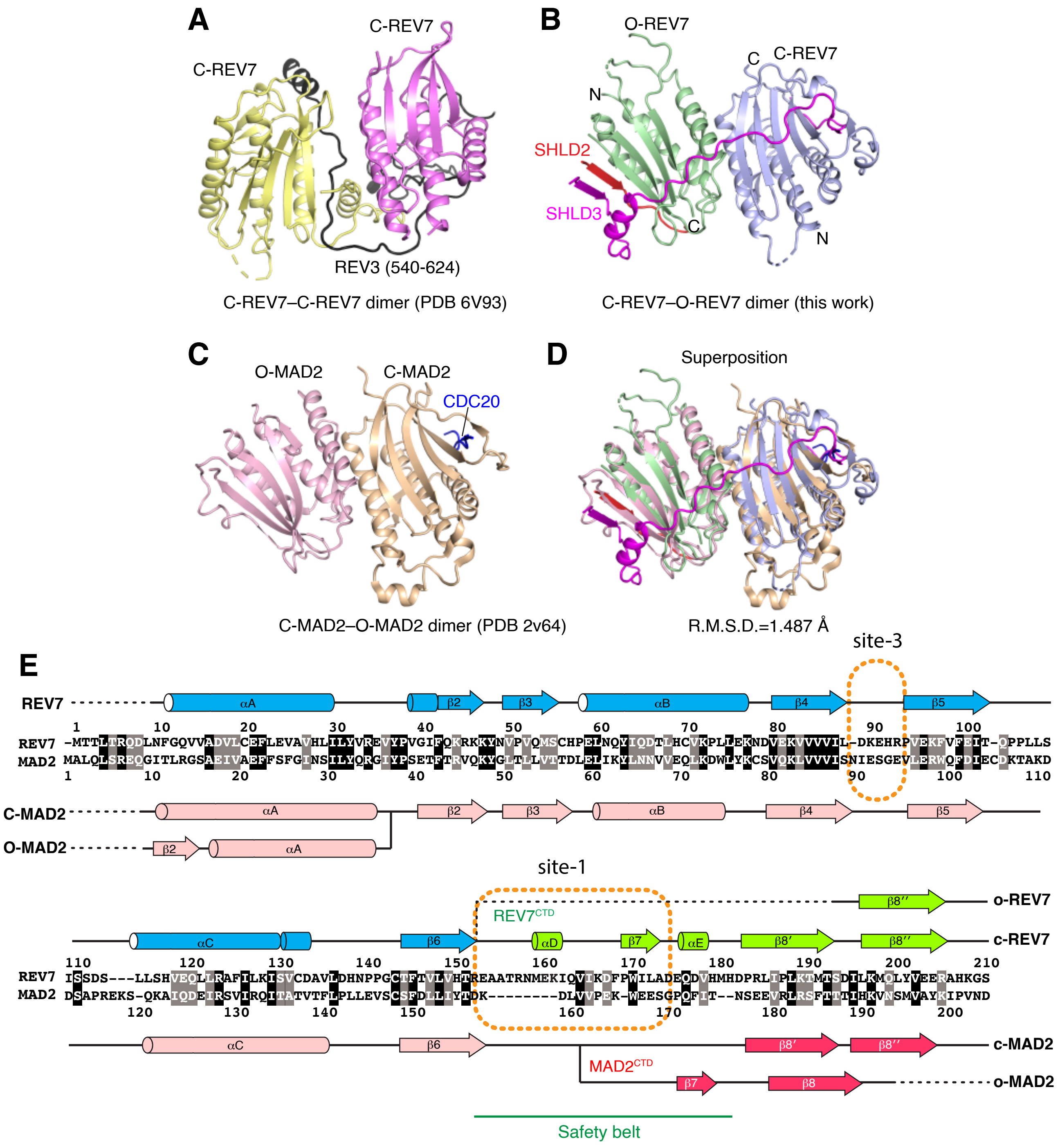

**Table S1. X-ray data collection and refinement statistics.**		
Sample	SHLD3s–REV7 (PDB 6WW9)	SHLD2.3–REV7$_4$ (PDB 6WWA)
**Data collection**		
Wavelength (Å)	0.9792	0.9792
Space group	*P* 4_3 2_1 2	*I* 4 3 2
Cell dimensions		
a, b, c (Å)	75.7, 75.7, 220.1	331.84, 331.84, 331.84
α, β, γ (°)	90, 90, 90	90, 90, 90
Resolution (Å)	29.04- 2.70 (2.80-2.70)	39.11-3.80 (3.94-3.80)
R-merge	0.165 (1.319)	0.403 (4.971)
I/σ*I*	8.87 (1.42)	10.30 (0.86)
Completeness (%)	97.8 (99.3)	99.7 (99.4)
Redundancy	4.3 (4.3)	35.8 (36.4)
CC1/2	0.991 (0.453)	0.999 (0.472)
Unique Reflections	18,113 (1,755)	30,856 (3,027)
**Refinement**		
R_{work}/R_{free} *(%)*	21.5/24.4	23.7/26.6
Reflections in refinement	18,011 (1,755)	30,823 (3,025)
No. of non-hydrogen atoms		
Macromolecules	3,543	6,776
Protein residues	434	835
B-factors ($Å^2$)		
Macromolecules	66.35	189.45
R.m.s. deviations		
Bond lengths (Å)	0.003	0.003
Bond angles (°)	0.53	0.70
Ramachandran plots		
Favored (%)	97.63	92.69
Allowed (%)	2.37	5.70

Outliers (%)	0	1.61
Statistics for the highest-resolution shell are shown in parenthesis.		

**Table S2. Cryo-EM data collection, processing, and validation statistics.**	
Sample	SHLD2.3–REV7$_4$–TRIP13(E253Q) (EMID-23244, PDB 7L9P)
**Data collection**	
Microscope	Titan Krios
Detector	Gatan K3
Automation software	SerialEM
Nominal magnification	22,500
Calibrated magnification	47,262
Voltage (kV)	300 kV
Total dose (e^-/Å^2)	53
Dose rate (e^-/pixel/s)	20
Number of frames collected	40
Defocus range (μm)	-1.0 to -2.5
Pixel size (Å)	1.064
Collected Micrographs	1,925
Selected Micrographs	1,363
**Reconstruction**	
Initially autopicked particles	1,212,927
Particles used for classification	846,918
Particles in the final map	104,023
Symmetry	C1
Resolution	
FSC 0.143 (unmasked/masked, Å)	3.7/3.6
FSC 0.5 (unmasked/masked, Å)	4.2/4.0
Map sharpening B factor (Å^2)	100
**Model composition**	
Protein	2919
Ligands	5
**Validation**	

MolProbity	2.34
Clash score	20.16
Map Correlation Coefficient	0.68
R.m.s. deviations	
Bond lengths (Å)	0.008
Bond angles (°)	1.238
Ramachandran plots	
Favored (%)	90.49
Allowed (%)	9.44
Outliers (%)	0.07
Rotamer outliers (%)	0.58
